# Influence of dust and sea-salt sandwich effect on precipitation chemistry over the Western Ghats during summer monsoon

**DOI:** 10.1038/s41598-019-55245-0

**Published:** 2019-12-16

**Authors:** L. Yang, S. Mukherjee, G. Pandithurai, V. Waghmare, P. D. Safai

**Affiliations:** 1 Indian Institute of Tropical Meteorology, Pune, India; 2 Savitribai Phule Pune University, Pune, India

**Keywords:** Climate sciences, Atmospheric science, Atmospheric chemistry

## Abstract

Assessment of Sea Salt (SS) and Non-Sea Salt (NSS) aerosols in rainwater is important to understand the characterization of marine and continental aerosols and their source pathways. Sea salt quantification based on standard seawater ratios are primarily constrained with high uncertainty with its own limitations. Here, by the novelty of *k*-mean clustering and Positive Matrix Factorization (PMF) analysis, we segregate the air masses into two distinct clusters (oceanic and continental) during summer monsoon period signifying the complex intermingle of sources that act concomitantly. The rainwater composition during strong south-westerly wind regimes (cluster 2-oceanic) was profoundly linked with high sea salt and dust, whereas north-westerly low wind regimes (cluster 1-continental) showed an increase in SO_4_^2−^ and NO_3_^−^. However, SO_4_^2−^ abundance over NO_3_^−^ in rain-water depicted its importance as a major acidifying ion at the region. The satellite-based observations indicate the presence of mid-tropospheric dust at the top (3–5 km) and marine sea salt at bottom acts as a “sandwich effect” for maritime clouds that leads to elevated Ca^2+^, Na^+^, Mg^2+^, and Cl^−^ in rainwater. This characteristic feature is unique as sea spray generation due to high surface winds and dust aloft is only seen during this period. Furthermore, four source factors (secondary inorganic aerosol, mixed dust & sea salt, biomass burning & fertilizer use, and calcium neutralization) derived from PMF analysis showed contribution from local activities as well as long-range transport as dominant sources for the rainwater species.

## Introduction

Sea salt contributes to the major fraction of aerosol population over the marine boundary layer and it is usually generated over the oceans via winds. These momentous component of aerosol particles are predominantly produced through wave break-action which entrains air into the sub surface ocean depth that rise up back to the ocean surface as bubbles causing white caps and burst^1^, thus injecting seawater drops into the atmosphere. Wind speed plays a crucial part in triggering the waves whereas wind direction and turbulent mixing helps in entraining the sea salt spray into the atmosphere. Also, the rate of interfacial sea salt (sea salt generated at ocean surface) and effective sea salt (sea salt that sustains in the atmosphere for longer period of time) production is essentially governed by conditions such as humidity, wind, and dry depositional rates^2^. The net loss of the sea salt is mostly through gravitational settling and wet deposition in the coarse mode regime^3^; where it tends to fall down under the force of gravity back to the ocean. Therefore, small drops/fine mode particles efficiently entrain vertically into the atmosphere.

Sea salt also scatters the solar radiation because of strong refractive index and can attain near to unity single scattering albedo^4^. On the other hand, it can also act as an efficient hygroscopic cloud condensation nuclei that elevate the water uptake to form clouds and can affect net radiative balance via radiative cooling^5–8^. The quantification of sea salt (SS) and non-sea salt (NSS) aerosol source contribution in precipitation over land and ocean is important to understand their complex intermingle chemistry^9–11^, and their pathways for dominant aerosol regimes that may affect pH of rainwater^12–14^ and subsequent quantification of natural and anthropogenic sources of sea and non-sea originating aerosol species.

Summer monsoon or South West (SW) monsoon brings on an average 800–1000 mm of orographic rainfall^15^ over the Western Ghats accompanied by warm low level maritime cloud that remains of prime time to understand the loading of water-soluble inorganic aerosol species in rainwater SS and NSS source apportionments. Seawater fractions have been used to estimate the SS and NSS sources of soluble inorganic species dissolved in rainwater over the past decades^16,17^. However, the methodology suffers from major limitations such as: firstly no fractionation/loss occurs while the formation, injection and transport of sea salt aerosols of cloud bearing species; and secondly all reference species are of sea salt origin^18^. But, as the monsoon clouds engulf over the orographic region of Western Ghats, the pollution from windward cities presides the fate of rainwater aerosol species as one may expect the signatures of sea salt to be lost in the course of time and path due to wash out or aqueous phase reactions. Therefore, this may tend to underestimate/overestimate the actual amount of NSS species in rainwater concentrations. In the present study, we utilize *k*-mean clustering algorithm^19–21^ imposed over hourly HYSPLIT (HYbrid Single-Particle Lagrangian Integrated Trajectory) backward (5 days) trajectories to separate discrete air masses originated during SW-monsoon period over the Arabian Sea reaching at the receptor site, Mahabaleshwar (High Altitude Cloud Physics Laboratory-HACPL 17.92° N, 73.66° E) at an altitude of 1375 m above mean sea level definite into two clusters to segregate as NSS and SS spells respectively. This cluster classification is applied to daily 113 rainwater samples during 2016 monsoon rainfall that is further based on greater than 60 percent threshold trajectories grouped into a specific cluster for a day.

## Results & Discussions

### Surface meteorological conditions over the Arabian Sea

**Figure 1:**
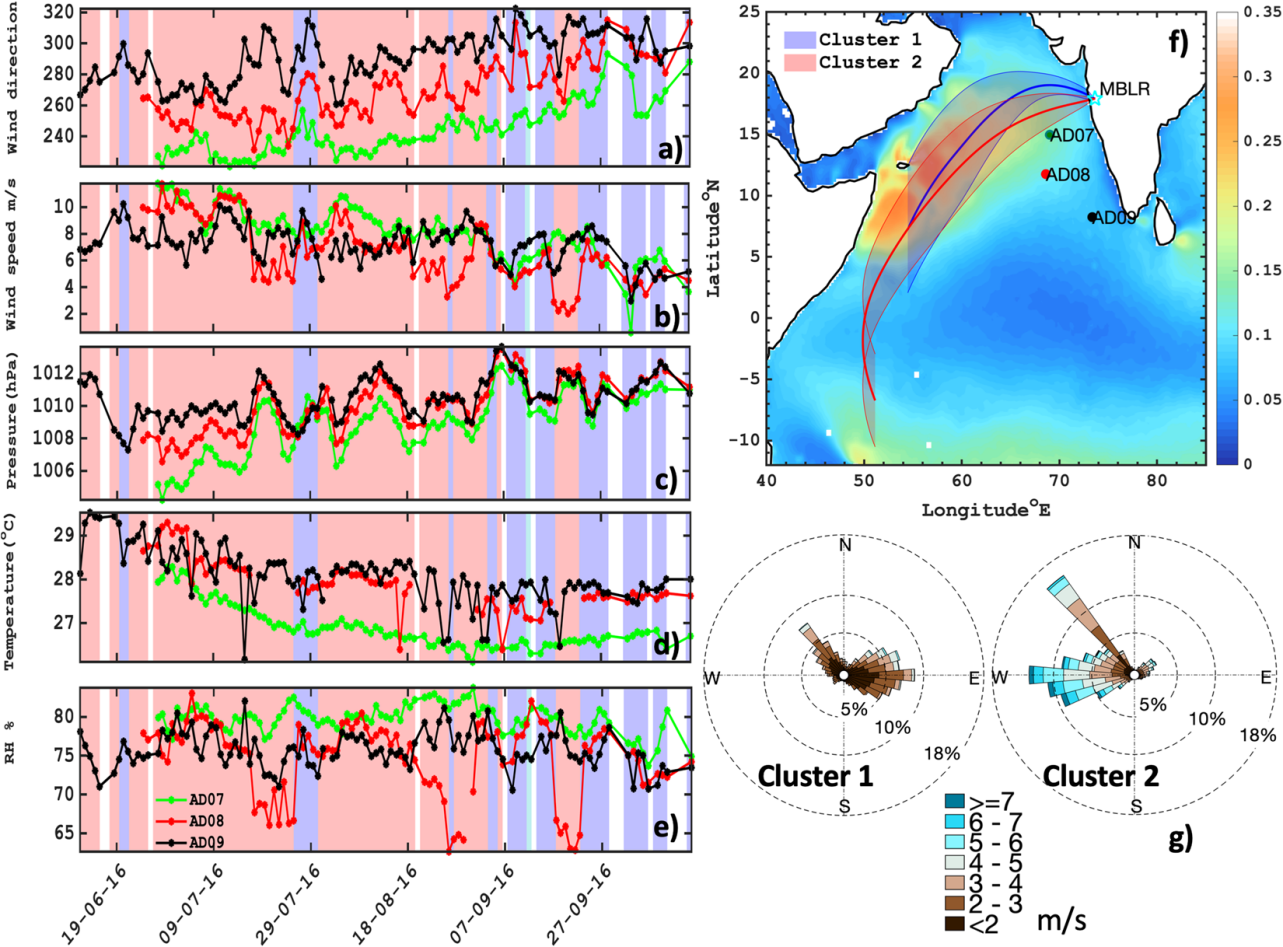
Daily mean time series of ocean surface meteorological parameters (wind speed- m/s, direction- deg°, pressure- hPa, ambient temperature- °C, and RH- %;) from OMNI Buoy (AD07, AD08, and AD09) dataset (**a**–**e**). The shaded region in blue, red and white represents cluster-1, cluster-2 and missing dates. Spatial map (**f**) of HYSPLIT clustered backward trajectories at Mahabaleshwar receptor site (MBLR), mapped over ASCAT ocean surface wind stress (Pa). And local AWS clustered wind rose diagram (**g**) at site location HACPL- Mahabaleshwar during summer monsoon 2016.

Figure 1(a–e) shows daily time series of latitudinally aligned *in-situ* OMNI buoy surface observation of meteorological parameters such as wind speed and direction, temperature, pressure and relative humidity over the Arabian Sea. AD09 buoy is situated in the south, AD07 in the north and AD08 in the intermediate coastal region as shown in Fig. 1f. It is inevitably seen that during the initial phase of monsoon, the latitudinal gradient of north-south surface pressure is prominent near to ~2 hPa. This shift in low pressure is well reflected by relatively stronger winds observed by the AD07 buoy. The near difference in latitudinal wind speed between AD07 to AD09 is ~1.5 m/s. And this stronger wind and cloudy regime is further found to cool the surface air and supports the viable change observed near to surface 2 m temperature (~1 °C between north-south gradient) across the Arabian Sea. The shift in prevailing wind direction during monsoon from south-west to north-east direction is well known. However, the latitudinal north-south shift in wind direction at the surface is quite unique and substantial difference is observed (~30–40°) between AD07 and AD09. This rarity is important in order to understand the long-range transport at coastal sites during rainwater collection.

The wind speed at the ocean surface is one of the major variables for generating the sea salt aerosols. On the other hand, wind direction is an equally important factor that controls the advection of these particles. Figure 1f shows averaged ASCAT satellite - wind stress (Pa-Pascal) over the Arabian Sea at 25 × 25 km spatial resolution during summer monsoon of 2016. The wind stress signifies the roughness of the ocean surface for generation of waves, which may lead to the initiation of sea salt spray. The maximum wind stress of ~0.3 Pa is well seen near to the Somali coast. However, gradient of wind stress was observed between AD07 to AD09 buoy that varied between ~0.25 to 0.1 Pa. This indicated strong wind force over the ocean surface for generation of ocean waves in northern region as compared to southern Arabian Sea, which may play a major role in sea spray generation. The areal proximity of Mahabaleshwar (MBLR) is closer to AD07, hence the prevailing conditions of sea salt generation at ocean surface near to AD07 should resonate in MBLR. However, during active and break phases of monsoon, the wind speed and directions highly fluctuate and may be of oceanic and continental origin. Hence, *k*-mean cluster algorithm is implemented to separate the wind variability into 2 clusters as shown in Fig. 1f. Cluster 1, is designated as the one most influenced by continental air masses with northerly components of slow winds (also see in Suppl. Fig. 1) whereas cluster 2 purely reflected dominance of oceanic monsoonal south westerly flows. This is also evident from Fig. 1g, as shown in the wind rose diagram from local Automated Weather Station (AWS) at MBLR. The cluster 1 wind rose reveals that the significant amount of winds reaching at site from north west-eastward direction with magnitude mostly constrained to below ~2–3 m/s. Whereas, cluster 2 mostly displayed strong south-westerly flows reaching up to ~4–7 m/s. This is also well validated by comparing the normalized probability and PDF(probability density function) of both the clusters (Suppl. Fig. 1), that is computed based on solely HYSPLIT backward trajectory at 100 m above ground level and independent segregation of local AWS data by *k*-mean cluster algorithm. The wind direction in cluster 1 predominantly exhibited north-easterly flows, besides some intersection of south-westerly winds and vice versa (Suppl. Fig. 1a,b). The higher spread of wind speed PDF in cluster 2 (Suppl. Fig. 1b,d) between 0 to 8 m/s depicted higher variability in magnitude as compared to cluster 1. In spite of it, the mean difference in wind speed of both the clusters is ~2.5 m/s.

### Cluster analysis of aerosol optical depth and wind

**Figure 2:**
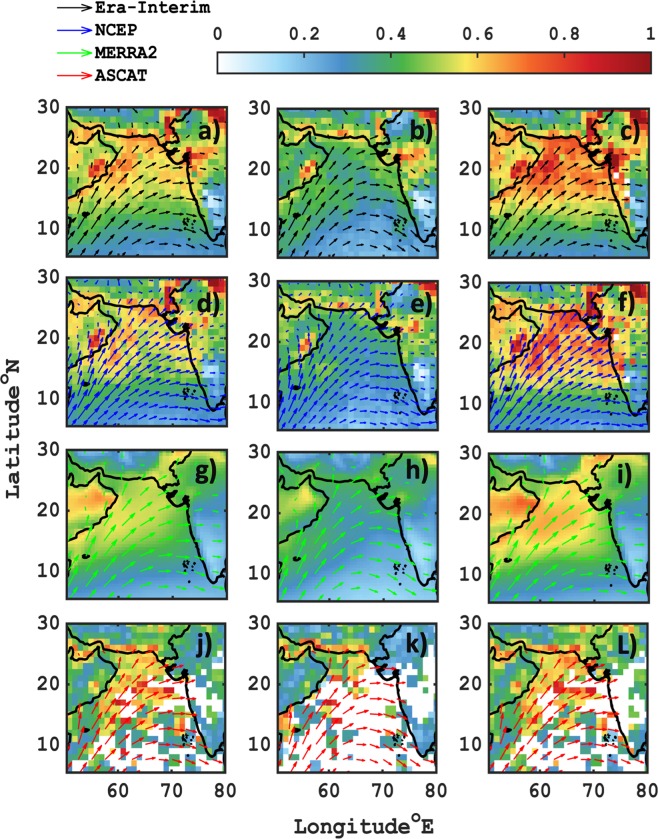
Spatial map of averaged AOD and wind direction over the Arabian sea, for Total (first column), cluster-1 (second column) and cluster-2 (third column). The first (**a**–**c**) and second row (**d**–**f**) represents MODIS-Terra and Aqua DB DT combined total AOD at 550 nm, overlaid by Era-Interim and NCEP reanalysis 10 m wind fields respectively. The third row (**g**–**i**) shows MERRA2 total AOD (550 nm) and its 10 m wind fields. And last row (**j**–**L**) corresponds to OMI-Aura total AOD at 500 nm superimposed by ASCAT METOP-A surface wind vectors.

The influence of wind on total Aerosol Optical Depth (AOD) measured over the Arabian Sea is further analyzed using satellite and reanalysis data products. Figure 2(a,d,g,j) shows average spatial pattern of columnar AOD and wind field over the Arabian Sea during south-westerly monsoon (113 rainwater collection days) period. The MODIS Deep Blue (DB) and Dark Target (DT) combined product of daily total AOD at 550 nm showed very high values of AOD that reached up to unity in cluster 2 dominated days (Fig. 2c,f), whereas in cluster 1, there were almost less than half (~0.4) (Fig. 2b,e). This increase in cluster 2 is attributed to the major fraction being contributed through the aloft wind-blown dust layer loading^14^ and surface wind-generated marine sea salt fractions within the boundary layer. A similar agreement was seen between total AOD at 500 nm derived from OMI satellite (Fig. 2k,L), and MERRA2 (Fig. 2h,i) at 550 nm. The average spatial pattern of mean AOD variability in all the satellite and reanalysis products exhibited spatial resemblance for cluster 1, 2 and average AOD (total). In addition to it, the standard deviations (see Suppl. Fig. 2) of mean values for total AOD across all the platforms were found to be significantly low (~0.1 for cluster 1 and ~0.25 for cluster 2). However, in spite of cloud screened level 3 MODIS DB DT product in cluster 2 showed anomalous standard deviation as high as 1 near to the coast of Oman, and Pakistan. This high value is attributed to sporadic strong Shamal winds that merge with low-level jet at ~850 hPa, thus uplifting hefty dust layer across the ocean. This indicates that the strength (Suppl. Fig. 3) and direction of wind-blown aerosol substantially governs the columnar aerosol loading which in turn may also influence the rainwater concentration.

The ASCAT obtained ocean surface wind speed was also found to be in good accord with ERA-Interim, NCEP and MERRA2 reanalysis (Suppl. Fig. 3) products. The maximum wind reached up to ~15 m/s in cluster 2 across the Arabian Sea basin (Suppl. Fig. 3c,f,i,l) and was found very conducive for sea spray initiation. However, the winds in cluster 1 (Fig. 2b,e,h,k) showed drastic weakening (Suppl. Fig. 3b,e,h,k) of strength and a slight shift in direction from south-westerly to north westerly towards the West Coast of Indian sub-continent was observed. This change in slight direction was earlier reported to modulate the rainwater concentration and chemistry drastically, due to the major metropolis that falls in the upwind direction to the orographic feature^22^.

### Influence and generation of sea salt aerosol

**Figure 3:**
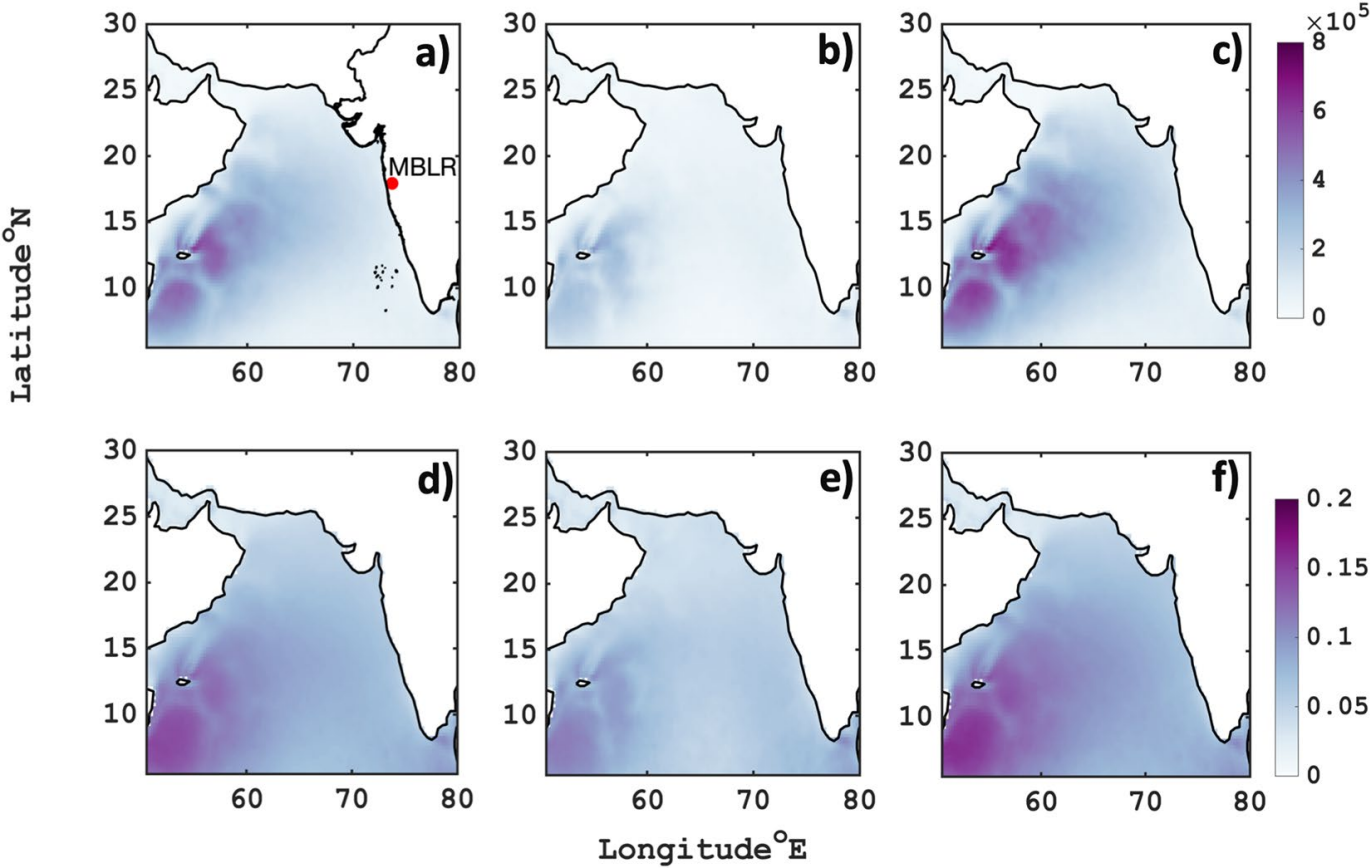
Spatial distribution of sea salt mass flux generation due to surface winds (total- **a**, cluster1- **b** and cluster2- **c**) by Gong 2003^2^ approach. And Vinoj & Satheesh 2004^23^ empirically wind derived sea salt AOD (total- **d**, cluster- **e**, and cluster2- **f**). The red dot depicts Mahabaleshwar-MBLR receptor location.

In order to understand the contribution of sea salt aerosol to rainwater, we first compute average sea salt fluxes (Fig. 3a) and sea salt AOD (Fig. 3d) over the Arabian Sea for 2016 monsoon. The flux is defined as the rate of sea spray generation per unit area of sea surface per increment of particle radius. The sea salt source function parametrization scheme is based on empirical relationship between wind speed and shape factor^2^. The wind speed obtained from ASCAT is well validated with daily buoy *in situ* measurements (Suppl. Fig. 4). The spatial pattern (as shown in Fig. 3c) of ASCAT derived sea salt fluxes clearly indicates an excessive amount of sea salt droplet production in cluster 2 (~8 × 10^5^ m^−2^μm^−1^ s^−1^) and covers most of the Arabian Sea basin. On the other hand, cluster 1 (Fig. 3b) indicates of very low amount of sea salt fluxes (~1 × 10^5^ m^−2^μm^−1^ s^−1^). Moreover, based on wind index and calm wind state, an empirically derived relation for sea salt AOD^23^ varying latitudinally is also shown in Fig. 3b,c. The similar average spatial distribution of sea salt AOD is captured. The average sea salt AOD in cluster 1 (Fig. 3e) and 2 (Fig. 3f) are profoundly observed as near moderate (~0.05) to high (~0.2) in the east of the Arabian Sea respectively. The reason behind for secluded sea salt aerosol generation in east Somali coast between 5–20° N is because of intense cross-equatorial flow and surface winds during monsoon that is generally implicated in empirically derived fluxes and AOD. However, the source function for the amount of sea salt released into the ambient atmosphere does not account for the sink/loss and advection term. Thus, transport and transformation of sea salt are not ascertained by this fact but it essentially gives us vital information about sea salt production in cluster segregation. Hence, it is important to discern the fate of sea salt and other major aerosols (dust and sulphate) that includes source, sink and advection tendencies. Therefore, we explored NASA-MERRA2 and ECMWF-CAMS reanalysis products to ascertain the influence of different sources on the estimation of sea salt concentrations around the observation site.

### Transport of dust, sea salt and sulphate

**Figure 4:**
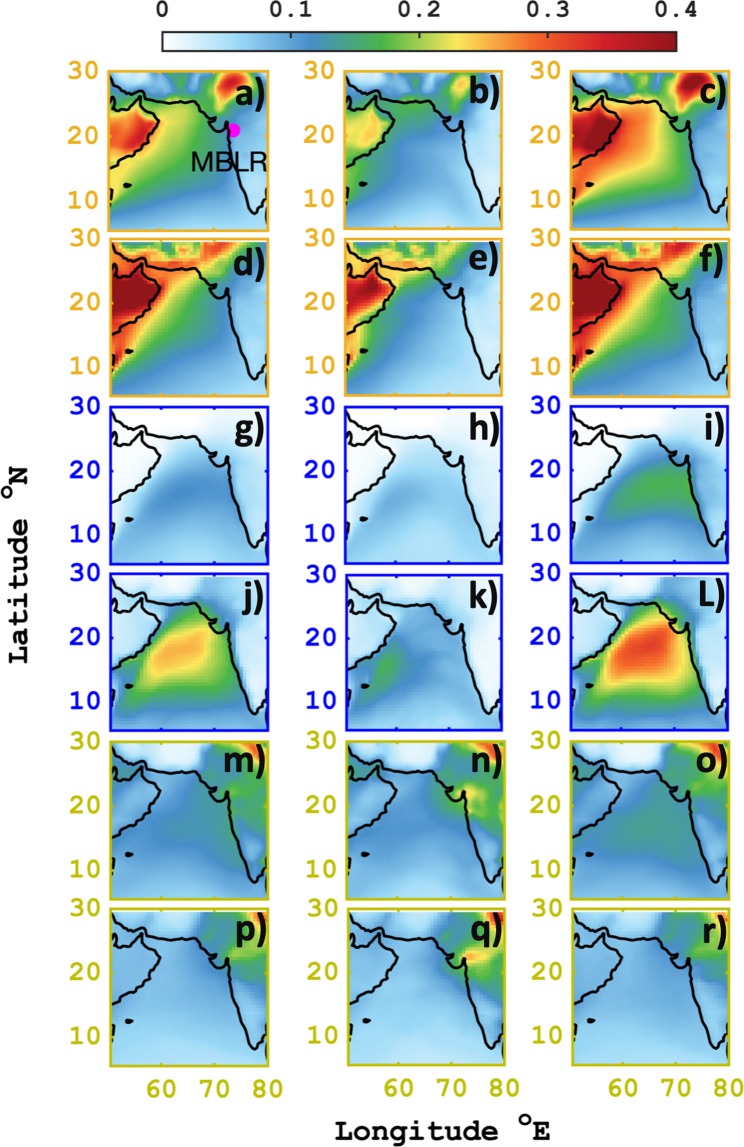
Spatial pattern of dust, sea salt and sulphate aerosol total extinction AOD at 550 nm from MERRA2 (dust **a**–**c**; sea salt **g**–**i**; sulphate **m**–**o**) and CAMS (dust **d**–**f**; sea salt **j**–**L**; sulphate **p**–**r**) reanalysis. The yellow frame outline represents dust, blue outline depicts sea salt and light green outline indicates sulphate AOD for Total (first column), cluster-1 (second column) and cluster-2 (third column).

Monsoon is a complex intermingling period of numerous sources that affect the rainwater and aerosol composition concomitantly. Figure 4(a,d,g,j,m,p) represents the average spatial pattern of dust, sea salt, and sulphate AOD at 550 nm respectively. The water-soluble inorganic daily rainwater concentration was noticed with highest percentage (~20–30%) contribution from Na^+^, Cl^−^ and Ca^2+^ (Suppl. Fig. 5a). The Ca^2+^ in rainwater is mostly contributed from mineral soil dust or from the sea salt in the form of CaCl_2_. These high percentages of Ca^2+^ were observed in both the rainwater cluster (1 and 2) samples. MERRA2 fine mode fraction (PM2.5 AOD**/**Total AOD) of dust and sea salt exhibited the presence of ~50% of finer dust (Suppl. Fig. 6a–c) and sea salt (Suppl. Fig. 6d–f) in both the clusters. However, in rainwater cluster 2, pre-dominance of Ca^2+^ to Na^+^ ratio (Suppl. Fig. 5b) was observed for most of the period with values extending beyond 1 and exorbitantly reached as high as ~5. Hence, such high values of Ca^2+^ are not expected in rainwater solely from sea salt.

Hence, Saharan dust is an important crustal source of metals and non-metals such as Al^3+^, Fe^3+^, PO^3−^, Pb^2+^, Mg^2+^, and Ca^2+^ found in rainwater^14,24^. And during monsoon season, cluster 2 (as shown in Fig. 4c,f) displayed inflated values of dust as high as ~0.4 all across the Arabian Sea. This enormity of dust is viewed as a clear signature of strong mid-tropospheric south westerly flow, that carries wind-blown dust from across middle east countries to Indian sub-continent blending in with moisture-laden maritime clouds that precipitate at the observation site. Also, in cluster 1, mineral dust (Fig. 4b,e) was distinctly isolated to land mass region near Oman, United Arab Emirates and Saudi Arabia due to weakening of the winds, thus hampering its long-range transport. Both MERRA2 and CAMS showed consensus in capturing the spatial variability of AOD but latter weighted on the higher bias side.

**Figure 5:**
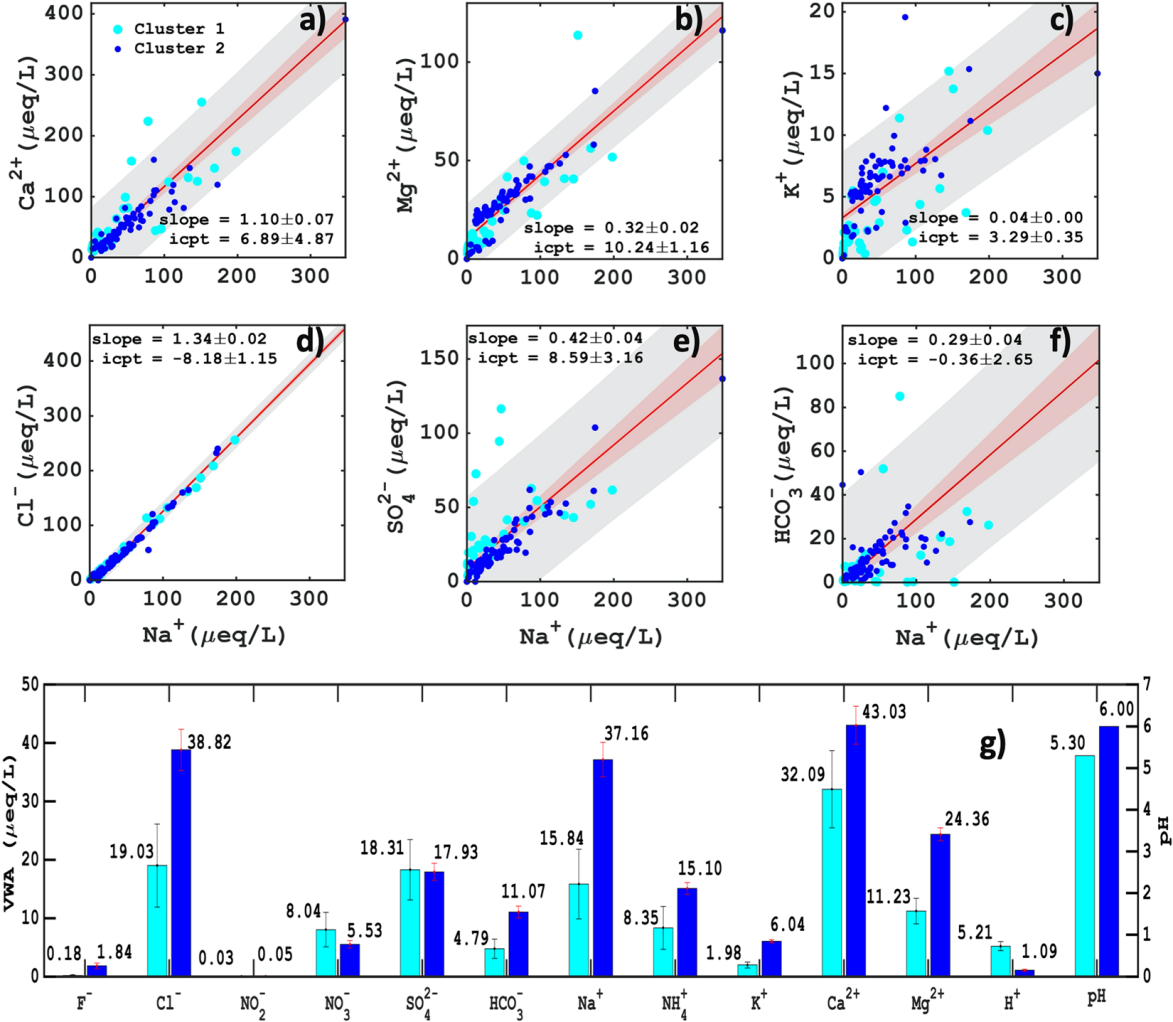
Scatter plot of ionic rainwater concentration of Na^+^ (as reference species) vs cations (**a**–**c**) and Na^+^ vs anions (**d**–**f**) in microequivalents per liter for 113 rainwater samples collected during 2016 monsoon rainfall at site HACPL, Mahabaleshwar. The grey shaded region signifies 95% prediction interval and red region indicates standard error of the slope. And volume-weighted average concentration with their weighted standard error^55^ **(g)** and pH, for all anions and cations in cluster-1 (cyan) and cluster-2 (blue).

The influence of long-range transported dust at mid-troposphere and near surface sea salt contribution to cloud is a unique association of aerosols that pertains during monsoon. Therefore, this complexity is further explored. Sea salt aerosols are found to be the most dominant species over the Arabian Sea in cluster 2 (Fig. 4i,l) as compared to cluster 1 (Fig. 4h,k) after dust. MERRA2 sea salt AOD values were found to be ~0.2, whereas CAMS AOD were as high as >0.4. The biases between MERRA2 and CAMS reanalysis are inherent features of the model that may be due to different modelling architecture and emission inventories. However, our focus here is to investigate whether the two state of the art data assimilation mimic the signals of aerosol properties in our clusters or not. Indeed it does. The percentage amount of SO_4_^2−^ aerosol present in the rainwater samples (Suppl. Fig. 5a) indicates low (~10%) daily percentage contribution except for cluster 1 followed days at departure monsoon period, where it rose up to ~30%. This increment and presence of sulphate AOD originating over the ocean is found to be as low as <0.1. Moreover, pronounced high sulphate AOD (Fig. 4n,o,q,r) can be seen over the land as relative to the ocean, this rise is majorly due to anthropogenic activities that include mostly industrial and anthropogenic emissions on the windward sides of the Western Ghats^25^ during the season. The elevated sulphate AOD (>0.3) visible in cluster 1 (Fig. 4n,q) north-westward to MBLR confirms this finding and its presence in rainwater with a slight increase in rainwater SO_4_^2−^ and NO_3_^−^ concentration (Fig. 5g) is affirmed. It was also noticed that NSS sulphate (non-sea origin) was major contributor to rainwater SO_4_^2−^ and was found to dominate (~80%) in both the clusters, indicating highest fractional contribution from land whereas only ~20% from SS^26^ (shown in Suppl. Fig. 7), which also justifies for very low sulphate AOD observed over the Arabian Sea.

### *In-situ* measurements of rainwater species

The seawater ratios have been extensively used and still continue to be the important way of source apportionment to separate out the NSS and SS fraction of aerosol present in precipitated rainwater. However, our findings indicate that dilution of polluted continental air masses (from inland and long-range transport) would deviate the seawater ratios from the widely used methods with total assumption of Na^+^ and Mg^2+^ as reference species (see Table 1). One of the pathways is when unreacted sea salt-NaCl of oceanic origin reacts with the landmass pollutants such as; NSS SO_4_^2−^, NO_3_^−^ and organic acids to replace the Cl^−^ ions that may lead to Cl^−^ depletion in rainwater sample. It was observed that Cl^−^ % depletion was found to be ~30% and reached as high as ~50% at peak rainy period in cluster 2 (see Suppl. Fig. 8a) during monsoon. On the other hand cluster 1 showed very low ~5% depletion. The Cl^−^ depletion occurs through two main processes firstly when NO_3_^−^ reacts with NaCl to form NaNO_3_ and Cl^−^ (gas)^27^; and secondly where NSS SO_4_^2−^ reacts with NaCl to form Na_2_SO_4_ and Cl^−^ (gas)^28^. Thus in an ideal condition of non-reacting NaCl of oceanic source the ratio of Na^+^/Cl^−^ should be ~0.85 (see Suppl. Fig. 9a). However, Na^+^/Cl^−^ ratios for cluster 2 were mostly found to be >1 giving evidence that Cl^−^ had depleted. Hence, under the assumption that parts of depleting Cl^−^ may be due to NSS SO_4_^2−^ or NO_3_^−^ chloride depletion has been computed^9^ (see Suppl. Fig. 8) and surprisingly we saw (see Suppl. Fig. 9b) the number (~25) of rainwater samples having ratios of Na^+^/(NO_3_^−^ + Cl^−^) in cluster 2 was much higher than Na^+^ /(NSS SO_4_^2−^ + Cl^−^) (see Suppl. Fig. 9), suggesting most of the Cl^−^ depletion was due to NSS SO_4_^2−^. This was also validated by correlation coefficient analysis. For cluster 2 SO_4_^2−^ and Na^+^ were found to be very strongly correlated (R~0.95 - see Table 1), whereas the correlation between NO_3_^−^ and Na^+^ was ~0.7. Surprisingly, for cluster 1 both SO_4_^2−^ and NO_3_^−^ were moderately correlated with Na^+^ (R~0.5). Therefore under these depleting regime seawater ratios should be cautiously used. It is also good to mention the depletion of Cl^−^ was found to be higher in fine mode regime as compared to coarse mode, which is mostly related to their larger surface area distribution and longer atmospheric residence time^9^.

**Table 1:**
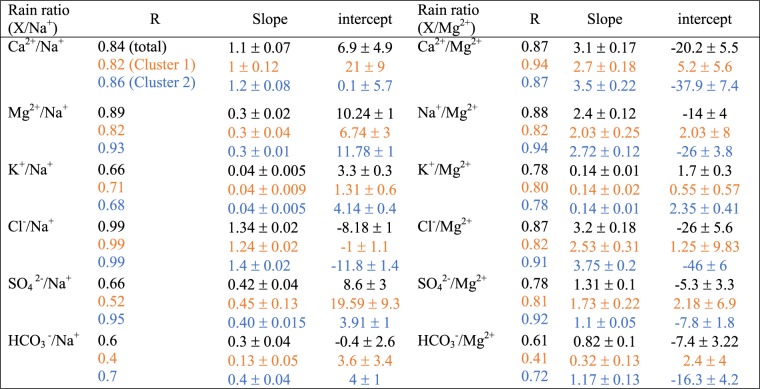
Mahabaleshwar rainwater compositional linear regression and Pearson correlational analysis of cluster 1, 2 and total days.

^*^Note: All values shown above are for year 2016 summert monsoon. Here, cluster 1 includes - 38 days, cluster 2–75 days; and Total -113 days.

As shown in Fig. 5(a–f), the cluster 1 rainwater species (Ca^2+^, Mg^2+^, K^+^, SO_4_^2−^, HCO_3_^−^) mostly fell out of the 95% prediction interval of total rainwater sample regression analysis and is presumably affected by continental air masses. Whereas in cluster 2, it was mostly seen to (Fig. 5a–f) fall within the intervals. The Ca^2+^/Na^+^ total slope value was found to be 25 times higher than the actual reported seawater ratios in the literature (shown in Table 2), which implies the existence of strong dust mineral (Ca^2+^) dissolution in rainwater samples. Similarly, cluster 2 and 1 displayed 28 and 21 times higher values respectively. We explicitly found that our rainwater regression slope value nowhere comes close to seawater ratios (see Table 2). However, clustered volume-weighted average (VWA) depicted a bit high values of NO_3_^−^ and SO_4_^2−^ as 5.5 and 0.38 μequivalent/liter in cluster 1 as compared to cluster 2, this supports and shows that the anthropogenic intervention in rainwater from local inland air masses are evident. This desecration of sea salt in rainwater is due to numerous factors acting concurrently such as local anthropogenic sources, long-range transport, sea salt transformation in aqueous phase chemistry with other aerosols, which might lead to loss of sea salt fraction in due course of time and passageway to inland precipitation. Subsequently, VWA for Ca^2+^, Mg^2+^, Na^+^, and Cl^−^ were highest amongst all the other rainwater ionic species. More importantly Na^+^, Cl^−^, Ca^2+^, and Mg^2+^ were 15.84, 19.03, 32.09 and 11.23 μequivalent/liter in cluster 1 and twice as much high in cluster 2 (37.16, 38.82, 43.03 and 24.36 μequivalent/liter) except for Ca^2+^, which was present in high amount in both the clusters. Despite pre-dominance of Ca^2+^ present in both the clusters further decrease in pH value (as seen in Fig. 5g) in cluster-1 (pH-5.3) confirmed an increase in anions majorly contributed by NO_3_^−^ and NSS SO_4_^2−^ (Suppl. Fig. 7) of long-range transported origins^29^, as compared to cluster-2 (pH-6) with less acidic in nature. The presence of dust and marine aerosol round the duration near to the Western Ghats region was also validated with CALIPSO LiDar aerosol classification image (Suppl. Fig. 10). The snapshot of CALIPSO images at initial, mid and final phase of monsoon 2016, clearly showed the layer of marine aerosol overlaid by dust layer in the mid-troposphere persisted throughout the period.

**Table 2:** Comparison between slope values of cluster 1, 2, and total rainwater constituents with bulk seawater ratios.

Rainwater ratios	Cluster 1	Cluster 2	Total	Seawater ratios (Wilson 1975)
Ca^2+^/Na^+^	0.93	1.18	1.1	0.044
Mg^2+^/Na^+^	0.33	0.32	0.32	0.228
K^+^/Na^+^	0.04	0.042	0.04	0.0218
Cl^−^/Na^+^	1.24	1.4	1.34	1.17
SO_4_^2−^/Na^+^	0.45	0.40	0.42	0.121
HCO_3_^−^/Na^+^	0.127	0.37	0.3	0.00508
Ca^2+^/Mg^2+^	2.7	3.5	3.1	0.194
Na^+^/Mg^2+^	2.03	2.72	2.4	4.40
K^+^/Mg^2+^	0.137	0.138	0.14	0.0958
Cl^−^/Mg^2+^	2.54	3.75	3.2	5.13
SO_4_^2−^/Mg^2+^	1.73	1.14	1.31	0.530
HCO_3_^−^/Mg^2+^	0.32	1.16	0.82	0.0223

All values are derived from concentration in rainwater sample in units of μequivalent/liter. And are significant at 95% confidence intervals at p-value < 0.05 (two-tailed t-test).

### PMF and local source apportionment

**Figure 6:**
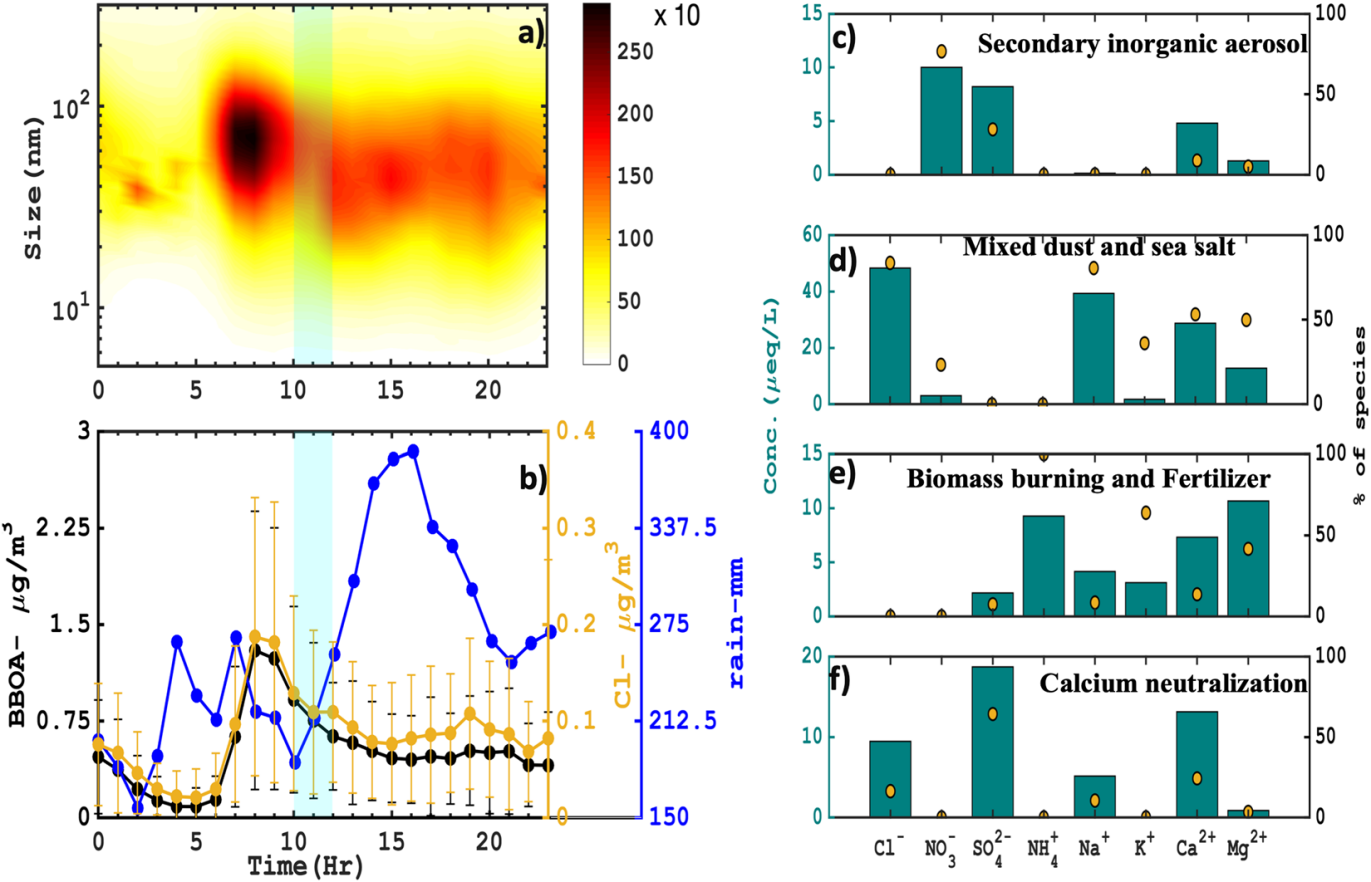
Diurnal hourly variation of **(a)** number size distribution(cm^−3^), and **(b)** BBOA (Biomass Burning Oxygenated Aerosol), non-refractory chloride mass concentration (μg/m^3^) along with net rain amount (mm) at site HACPL, Mahabaleshwar during 2016 summer monsoon rainfall. The cyan shade represents tentative time period for daily APC rainwater sample retrieval after 24 hrs collection. The PMF factor profiles of 4 factors and their respective rainwater ionic concentration and percentage contribution to each factor (**c**–**f**).

Further to ascertain the influence of different sources on rainwater composition, Positive Matrix Factorization (PMF; EPA-PMF 5.0)^30,31^ analysis was carried out. PMF is a multivariate bilinear model that deconvolutes a sample matrix data into a factor contribution and factor profile matrix. The uncertainty associated with the measurement was constructed following the approach by Anttila, 1995^32^. PMF analysis was performed up to 9 factors and the optimum 4-factor solutions (Fig. 6c–f) was chosen on the basis of scaled residuals^33,34^ and factor profiles physical resemblances with the regional sources, also discussed in Suppl. Fig. 11. As can be seen from Fig. 6c, Factor 1 majorly comprised of SO_4_^2−^ and NO_3_^−^ ion with 28% and 77% contribution respectively indicated of secondary inorganic aerosol source factor. On the other hand Factor 2 (Fig. 6d) mostly signified a combination of two different sources, that is wind-induced sea salt and long-range transported mineral dust mostly linked with characteristic ions such as: Na^+^ (81%), Cl^−^ (84%), Ca^2+^ (53%), and Mg^2+^ (50%). Moreover, Factor 3 (Fig. 6e) majorly constituted of K^+^ (64%), NH_4_^+^ (100%) and Mg^2+^ (42%), mostly attributed to biomass burning and fertilizer sources active during the season. In addition to it, the diurnal variation of biomass burning organic aerosol (BBOA, derived from the PMF analysis on PM1 aerosol), and non-refractory Cl^−^ from Aerosol Chemical Speciation Monitor exhibited morning hours (7–10 hr) peak high mass concentration at ~1.4 and ~0.2 μg m^−3^ respectively (shown Fig. 6b). This suggested a significant contribution of biomass burning emission to the ambient air^34^. Also, recent study^34,35^ for the same region had also documented the impact of wood burning on the aerosol number size as well as mass variability during the monsoon period. This type of freshly emitted particles was solely found to dominate in 70–100 nm particle size range and was recorded by the collocated scanning mobility particle sizer instrument, as can be seen in Fig. 6a the number concentration reached as high as ~2500 cm^−3^. The cyan window (Fig. 6b) indicates the automatic precipitation collector rainwater sample retrieval time and disdrometer net rain (mm) in those periods further suggest the encapsulation of these burning episodes in rainwater (due to scavenging) that may have inflict the rainwater concentration. Moreover the existence of Mg^2+^ along with K^+^, and NH_4_^+^ in same factor 3 was linked to agriculture fertilizer used for farming in this region. Ultimately Factor 4 is attributed as acid neutralization factor. Here in absence of NO_3_^−^ from factor 4 (shown & discussed in Suppl. Fig. 12) suggest Ca^2+^ (24%) acted as an important neutralizing agent for SO_4_^2−^ (64%) ion in the atmosphere. The abundance (~2–4 times higher) of SO_4_^2−^ (see Fig. 5g) over NO_3_^−^ in rainwater implied the availability of the SO_4_^2−^ ions in neutralisation process is way much higher as compared to the NO_3_^−^ ions. As also seen in Suppl. Fig. 10, dust mostly prevailed regionally. Sulfur dioxide (SO_2_) emitted from the combustion sources (biomass or fossil fuel burning) may have in turn underwent heterogeneous oxidation and subsequently got neutralized by Ca^2+^ ion present in the cloud droplets. It is also important to mention that the heterogeneous phase uptake and oxidation pathways of SO_2_ are much faster^36^ than NO_x._. And few studies have reported the basic nature of rainwater in this region with immense presence of Ca^2+^ in higher concentration^37^. This availability of Ca^2+^ in the cloud water fabricates more conducive environment for the heterogeneous uptake of SO_2_.

## Conclusions

The present study utilizes rainwater composition data from June to October 2016 at Mahabaleshwar, a high altitude site situated in Western Ghats mountain region in peninsular India along with various other available satellite and reanalysis datasets to assess the impact of different sources on the presence of major inorganic water-soluble constituents of the rainwater. *k*-mean clustering algorithm was primarily applied to air mass back trajectories to segregate the point observation as well as satellite and model reanalysis datasets. Cluster analysis reveals that the region is mostly influenced by two types of clusters during south-west monsoon, one with more of continental influence (cluster 1) and another with oceanic influence (cluster 2). The cluster wise calculated sea salt source function unveils the higher sea salt generation associated with cluster 2 as compared to cluster 1. Similarly, the presence of dust is also found more in cluster 2 as evident from MERRA2 and CAMS reanalysis data. The identical scenario was also visualized from the rainwater composition data with higher volume-weighted average concentrations of Na^+^, Cl^−^, and Ca^2+^ in cluster 2 as compared to cluster 1. Dust aloft and sea salt beneath over the measurement region depicted a perfect sandwich like condition which provides a continuous source for the rain-water constituents that show the limitations of the seawater ratio signatures which are far from standard marine values. In contrast, the secondary species like SO_4_^2−^ and NO_3_^−^ showed reverse trend indicating the possible influence of anthropogenic emissions in cluster 1 as it travelled toward inland. Thus, the segregation of rainwater samples with respect to cluster could not improve the seawater ratios significantly. The studies further enlighten the limitation of using seawater ratios for calculating sea salt and non-sea salt concentration as most of the time it may overestimate the non-sea salt fractions. The present study outlines extensively how different sources add up to the rainwater chemistry and showed an alternative way of identification and representation of sources using *k*-mean clustering and positive matrix factorization analysis.

## Method and Data

### Air mass classification by *k*-mean clustering algorithm

In order to separate the strong sea salt dominating air masses as SS and NSS spells that should prominently reflect the signatures in rainwater. We incorporate *k*-mean clustering algorithm to group the HYSPLIT backward trajectories^38^ based on the minimization of Euclidean distance from the centroid with numerous iterations to converge to a final set of assigned clustered trajectories. The Euclidean distance and centroid convergence is computed as per the following equation.

1 $$\begin{array}{lll} & k=2, & [{k}_{1},{k}_{2}]\\ {C}_{1}=[{x}_{1},{y}_{1}], & {C}_{2}= & [{x}_{2},{y}_{2}]\end{array}$$

2 $${D}_{1}({x}_{o},{y}_{o})=\sqrt{{({x}_{o}-{x}_{1})}^{2}+{({y}_{o}-{y}_{1})}^{2}},{D}_{2}({x}_{o},{y}_{o})=\sqrt{{({x}_{o}-{x}_{2})}^{2}+{({y}_{o}-{y}_{2})}^{2}}$$

if *D*_1_ < D_2_, then re-iterated centroid for cluster

3 $${C}_{1}=[\frac{{x}_{o}+{x}_{1}}{2},\frac{{y}_{o}+{y}_{1}}{2}]$$

else D_2_ < D_1_, then re-iterated centroid for cluster

4 $${C}_{2}=[\frac{{x}_{o}+{x}_{2}}{2},\frac{{y}_{o}+{y}_{2}}{2}]$$

here, *k* is the number of assigned clusters, based on our best-case well separated 2 clusters (as shown in Suppl. Fig. 1a,b). Here *C*_1_ and *C*_2_ are randomly selected initial centroids of two clusters with position coordinates of [x_1_, y_1_] and [x_2_, y_2_] respectively. And [x_o_, y_o_] is the position coordinate for trajectory under scrutiny. Therefore, Euclidean distance is computed from two centroids for [x_o_, y_o_], and based on minimum distance D_1_ or D_2_ is assigned to its corresponding cluster *k*_1_ or *k*_2_. In addition to it, this minimum distance approach is further implemented for generating the new centroid as shown in Eqs. (3) and (4) for next Euclidean distance calculation. The number of re-iteration of centroid is done until two clusters converge minimizing the error distance and no further change in centroid is observed.

Zefir tool was utilized for performing cluster analysis^39^. At first, each trajectory is defined to be a cluster, which implies, for N trajectories, there will be N number of clusters. For the first iteration, for every combination of trajectory pairs, the cluster spatial variance (SPVAR) is calculated. SPVAR is defined as the sum of the squared distances between the endpoints of the cluster’s component trajectories and the mean of the trajectories in that cluster. Then the total spatial variance (TSV), (the sum of all SPVAR) is calculated. The pair of clusters combined are the ones with the lowest increase in total spatial variance. After the first iteration, the number of clusters is N-1.

5 $${\rm{TSV}}=\sum ({\rm{all}}\,{\rm{SPVAR}})$$

6 $${\rm{SPVAR}}=\sum ({\rm{all}}\,{\rm{trajectories}}\,{\rm{in}}\,{\rm{cluster}})\,[\sum ({\rm{all}}\,{\rm{trajectory}}\,{\rm{endpoints}})\,\{{\rm{D}}\ast {\rm{D}}\}]$$

Here, D is the distance between a trajectory endpoint and the corresponding cluster-mean endpoint. The iterations continue until the last two clusters are combined, resulting in N number of trajectories in one cluster. In the first few clustering iterations, the TSV increases rapidly, then the TSV increases slowly for much of the clustering (~constant rate), but at some point it again increases rapidly, indicating that the clusters being combined are not alike. This sudden change in TSV can be used as a tool to identify the optimum number of clusters^40^. The iterative step just before the large enhancement of TSV can be assigned as the optimum number of clusters. For the present study, 2 optimum clusters were estimated (Suppl. Fig. 13) and to gain the statistical robustness these estimations were based on total number of 65088 HYSPLIT backward trajectories (Global Data Assimilation System- GDAS meteorology at 0.5° × 0.5°- 24 ensemble, hourly) for 113 rainwater collection days.

### *In-situ* Meteorological and other data used

Based upon vector computation, local AWS wind speed and direction for receptor HACPL site at Mahabaleshwar at 1 min temporal resolution was decomposed into u(x_i_) and v(y_i_) components as per below Eq. (7) and later *k*-mean algorithm was employed to it in temporal span and used as validation for HYSPLIT separated clusters.

7 $${x}_{i}={U}_{i}.\,\sin \,{(\Theta )}_{i},\,{y}_{i}={U}_{i}.\,\cos \,{(\Theta )}_{i}$$

Here Θ_i_ and U_i_ are wind direction and speed at i^th^ time, similarly *x*_*i*_ and *y*_*i*_ are zonal-u and meridional-v components of wind.

Moreover, *in-situ* surface meteorological (Wind speed, direction, Relative Humidity-RH, Pressure and Temperature) data over the Arabian Sea during summer monsoon 2016 were obtained from OMNI (Ocean Moored buoy Network for north Indian ocean) buoy network, that is further used for validating satellite wind products. There are total 5 buoys deployed in Arabian Sea, and in the present study we have utilized the meteorological data of only 3 buoys (AD07-14.9°N, 69°E, AD08-11.7°N, 68.6°E and AD09-8.2°N, 73.3°E) because of discontinuous temporal coverage during the study period. The wind speed and direction sensor has a resolution of 0.1 m/s and 0.1° with an accuracy of ±2% and 1.5–4°. In addition, pressure, RH and temperature sensor has an accuracy of ±0.15 hPa, ±1% and ±0.3 °C respectively. A two-tier data processing is carried out to OMNI buoy datasets as quality control measure. These checks include value, range, position and time, stuck value, and spike tests. A detailed description of quality control for these data can be found at https://incois.gov.in/documents/argoQCmanuals/INCOIS-DMG-TR-01-2009.pdf.

Daily 24 hr. rainwater samples were collected at HACPL-site by an Automated Precipitation Collector (APC). Eigenbrodt NMO-191/E is an advance enclosed system precipitation collector with circular collection a surface area of 500 cm^2^. The raindrop impaction on the sensor opens up the lid and auto shut helps in preventing dry deposition during non-rainy periods. The water-soluble inorganic aerosol concentration in rainwater was analyzed by offline chromatography technique (Methrohm IC-850; Supp5&C4). The ionic balance of total anion equivalents was compared with total sum of cation equivalents in rainwater to validate the Ion Chromatography (IC), in order to obtain more accurate balance HCO_3_^−^ ions were theoretically calculated and adjusted for anion equivalents^41,42^. Subsequently, for quality control of analytical results; the measured and computed conductivities^43,44^ (μS cm^−1^) were compared (see Suppl. Fig. 14).

The diurnal variation of daily total rainfall amount (mm) was analyzed by RD-80 impact disdrometer, which has a sensor that generates an electric pulse by impaction of falling raindrop hit on the styrofoam cone diaphragm of 50 cm^2^, that is further related to raindrop size by Marshal Palmer distribution. The disdrometer measures raindrop size spectra between 0.3 to 5 mm in every 30 secs frequency interval with an accuracy of ±5% of measured drop size. The BBOA (Biomass Burning Organic Aerosol) and non-refractory chloride mass concentrations (μg m^−3^) were acquired from ToF (Time of Flight) ACSM (Aerosol Chemical Speciation Monitor). ACSM measures non-refractory aerosol mass concentration of Organics, SO_4_^2−^, NO_3_^−^, NH_4_^+^, and Cl^−^ in between 50 nm to 1 μm size range with 10 min sampling interval. And its respective sensitivities are 0.06, 0.006, 0.007, 0.06, and 0.003 μg m^−3^. Additionally, GRIMM-Scanning Mobility Particle Sizer and Environmental Dust Monitor-number concentration (cm^−3^) in size range between 5.14 nm to 37μm was also utilized in this study. The Sample flow rate of the instrument was maintained to 1.2 L/min, ±3% constant due self-regulation and reproducibility varied ±3% of total measuring range.

### Calculation of sea salt aerosol

We first estimate spatial sea salt flux density over the ocean by utilizing parametrization scheme of sea salt source function from Gong *et al*.^2^ as shown in Eqs. (8, 9). The daily ocean surface (10 m) wind speed during monsoon 2016, was retrieved from ASCAT (Advanced SCATterometer) on-board EUMETSAT METOP-A satellite at 25 × 25 km grid resolution. And it is assimilated into the source function to enumerate the sea salt fluxes. ASCAT is a vertically polarized active C-band radar that measures backscatter signals from the ocean surface with linearly transmitting signal at 5.225 GHz. The coverage is by two swaths in left and right to nadir; with two sets of three slant receiver antennae’s at ±45° broadsides. Various internal and external calibration checks along with quality control and validations^45^ are performed to ASCAT-A L2 wind speed to gain stable results^46,47^.

8 $$\frac{d{f}_{o}}{dr}=1.373\,{u}_{10}^{3.41}{r}^{-A}(1+0.057{r}^{3.45})\times {10}^{1.607{e}^{-{B}^{2}}}$$

9 $$A=4.7{(1+\Theta r)}^{-0.017{r}^{-1.44}},\,{\rm{and}}\,B=(0.433-logr)/0.433$$

The df_o_/dr (particles m^−2^s^−1^μm^−1^) is the density function of sea salt generation, where u_10_ is 10-m wind speed. A and B are exponential parameters with Θ as adjustable shape factor and r is the drop radius. In addition, we also compute wind-induced sea salt AOD based on simple exponential relationship from Vinoj & Satheesh, 2004^23^.

### Satellite and reanalysis products

To understand the other rain dominating aerosol compositions along with sea salt, we have innocuously examined aerosol optical depth from reanalysis and satellite products. The Modern-Era Retrospective analysis for Research and Applications version 2 (MERRA-2) is an enhanced version of NASA’s MERRA v1. The upgraded version includes better assimilation of meteorology and aerosol optical depth from the ground-based measurements and spaceborne remote sensing platforms^48^; the retrospective global data products are available since 1980 and are well validated with ground-based observations^49^. In the current study, we have used total, dust, sulphate and sea salt AOD at 550 nm from MERRA2 (M2T1NXAER) and Copernicus Atmosphere Monitoring Service (CAMS) reanalysis daily product. In addition to that, wind fields (M2T3NVASM) and fine mode fraction (PM2.5 AOD/Total AOD) of dust and sea salt is also obtained from MERRA2 reanalysis.

The European Centre for Medium-Range Weather Forecasts (ECMWF) CAMS reanalysis products is available for the period 2003–2017. The reanalysis product of atmospheric composition uses 4Dvar data assimilation technique and is notably assessed and validated on quarterly basis for stable output^50,51^. Along with it, we have utilized surface winds from ERA-interim and NCEP (National Centers for Environmental Prediction) reanalysis. The wind fields biases are proportional to altitude, that suggest low bias near to the surface^52–54^.

In addition, aerosol properties were obtained from numerous spaceborne polar-orbiting satellite sensors such as Ozone Monitoring Instrument (OMI- Aura), Moderate Resolution Imaging Spectroradiometer (MODIS-Terra, Aqua), and Cloud-Aerosol Lidar with Orthogonal Polarization (CALIOP) onboard CALIPSO (Cloud-Aerosol Lidar and Infrared Pathfinder Satellite Observation) satellite. The MOD08_D3 is a level 3 quality assured and cloud filtered combined dark target and deep blue AOD product at 550 nm for land and ocean. Apart from Terra, rest of them belongs to A-train satellite constellation. MODIS-Aqua product MYD08_D3 is similar to MOD08_D3 except for the same orbital track as OMI-Aura and Calipso and equatorial passing at 1:30 local time. We have also included OMAERUVd_003 an OMI cloud screened level 3 AOD product at 500 nm. Moreover, to differentiate between different species (dust, marine, and smoke) of aerosol; backscatter profiles and their feature aerosol classification images were obtained from CALIOP.

## Supplementary information

Supplementary information

## References

[1] Lewis & Schwartz. *Monograph Volumes Deep Interior: Mineral Physics Marginal Seas Present* (2004).

[2] Gong SL (2003). A parameterization of sea-salt aerosol source function for sub- and super-micron particles. Global Biogeochem. Cycles.

[3] Chate DM (2007). Scavenging of sea-salt aerosols by rain events over Arabian Sea during ARMEX. Atmos. Environ..

[4] Andrews E (2019). Overview of the NOAA/ESRL federated aerosol network. Bull. Am. Meteorol. Soc..

[5] Korhonen H, Carslaw KS, Romakkaniemi S (2010). Enhancement of marine cloud albedo via controlled sea spray injections: A global model study of the influence of emission rates, microphysics and transport. Atmos. Chem. Phys..

[6] Murphy DM (1998). Influence of sea-salt on aerosol radiative properties in the Southern Ocean marine boundary layer. Nature.

[7] Jones TA, Christopher SA (2008). Seasonal variation in satellite-derived effects of aerosols on clouds in the Arabian Sea. J. Geophys. Res. Atmos..

[8] Twomey S (1977). The Influence of Pollution on the Shortwave Albedo of Clouds. J. Atmos. Sci..

[9] Chatterjee Abhijit, Adak Anandamay, Singh Ajay K., Srivastava Manoj K., Ghosh Sanjay K., Tiwari Suresh, Devara Panuganti C. S., Raha Sibaji (2010). Aerosol Chemistry over a High Altitude Station at Northeastern Himalayas, India. PLoS ONE.

[10] Gupta D (2015). Hygroscopic properties of NaCl and NaNO3 mixture particles as reacted inorganic sea-salt aerosol surrogates. Atmos. Chem. Phys..

[11] Iizuka Y (2012). The rates of sea salt sulfatization in the atmosphere and surface snow of inland Antarctica. J. Geophys. Res. Atmos..

[12] Encinas D, Calzada I, Casado H (2004). Scavenging ratios in an urban area in the Spanish Basque Country. Aerosol Sci. Technol..

[13] Bertrand G, Celle-Jeanton H, Laj P, Rangognio J, Chazot G (2008). Rainfall chemistry: Long range transport versus below cloud scavenging. A two-year study at an inland station (Opme, France). J. Atmos. Chem..

[14] Ramaswamy V, Muraleedharan PM, Babu CP (2017). Mid-troposphere transport of Middle-East dust over the Arabian Sea and its effect on rainwater composition and sensitive ecosystems over India. Sci. Rep..

[15] Revadekar JV, Varikoden H, Murumkar PK, Ahmed SA (2018). Latitudinal variation in summer monsoon rainfall over Western Ghat of India and its association with global sea surface temperatures. Sci. Total Environ..

[16] Fitzgerald JW (1991). Marine aerosols: A review. Atmos. Environ. Part A. Gen. Top..

[17] Keene WC, Pszenny AAP, Galloway JN, Hawley ME (1986). Sea-salt corrections and interpretation of constituent ratios in marine precipitation. J. Geophys. Res..

[18] Galloway JN, Likens GE, Keene WC, Miller JM (1982). The composition of precipitation in remote areas of the world. J. Geophys. Res..

[19] Arthur D, Vassilvitskii S (2007). K-Means++: the Advantages of Careful Seeding. Proc ACM-SIAM Symp. Discret. algorithms..

[20] Lloyd S (1982). Least squares quantization in PCM. IEEE Trans. Inf. Theory.

[21] Glascoe, L. G., Glaser, R. E., Chin, H. S. & Loosmore, G. A. Regional-Scale Wind Field Classification Employing Cluster Analysis. *Lawrence Livermore Natl. Lab*. 1–4 (1990).

[22] Yang L., Pandithurai G., Chate D.M., Rao P.S.P., Waghmare V., Iyer U. (2019). Evidence of precedent wind role on controlling PM1 wet scavenging of aerosols during monsoon rain events. Atmospheric Environment.

[23] Vinoj V, Satheesh SK (2004). Direct and indirect radiative effects of sea-salt aerosols over Arabian Sea. Curr. Sci..

[24] Guieu, C., Loÿe-Pilot, M. D., Ridame, C. & Thomas, C. Chemical characterization of the Saharan dust end-member: Some biogeochemical implications for the western Mediterranean Sea. *J. Geophys. Res. Atmos*. **107** (2002).

[25] Ghude SD, Fadnavis S, Beig G, Polade SD, van der ARJ (2008). Detection of surface emission hot spots, trends, and seasonal cycle from satellite-retrieved NO_2_ over India. J. Geophys. Res. Atmos..

[26] Willey JD, Kiefer RH (1993). Atmospheric Deposition In Southeastern North Carolina: Composition And Quantity. J. Elisha Mitchell Sci. Soc..

[27] Pakkanen TA (1996). Study of formation of coarse particle nitrate aerosol. Atmos. Environ..

[28] McInnes LM, Covert DS, Quinn PK, Germani MS (1994). Measurements of chloride depletion and sulfur enrichment in individual sea-salt particles collected from the remote marine boundary layer. J. Geophys. Res..

[29] Willey J, Kiefer R (1990). A contrast in Winter Rainwater Composition: Maritime versus Continental Rain in Eastern North Carolina. Am. Meteorol. Soc..

[30] Paatero P (1997). Least squares formulation of robust non-negative factor analysis. Chemom. Intell. Lab. Syst..

[31] Paatero P, Eberly S, Brown SG, Norris GA (2014). Methods for estimating uncertainty in factor analytic solutions. Atmos. Meas. Tech..

[32] Anttila P, Paatero P, Tapper U, Järvinen O (1995). Source identification of bulk wet deposition in Finland by positive matrix factorization. Atmos. Environ..

[33] Brown SG, Eberly S, Paatero P, Norris GA (2015). Methods for estimating uncertainty in PMF solutions: Examples with ambient air and water quality data and guidance on reporting PMF results. Sci. Total Environ..

[34] Mukherjee S (2018). Seasonal variability in chemical composition and source apportionment of sub-micron aerosol over a high altitude site in Western Ghats, India. Atmos. Environ..

[35] Singla V, Mukherjee S, Kashikar AS, Safai PD, Pandithurai G (2019). Black carbon: source apportionment and its implications on CCN activity over a rural region in Western Ghats, India. Environ. Sci. Pollut. Res..

[36] Seinfeld, J. H. & Pandis, S. N. Chemistry of the atmospheric aqueous phase. *Atmos. Chem. Phys. From Air Pollut. to Clim. Chang*. **337407** (1998).

[37] Momin GA (2005). Study of chemical composition of rainwater at an urban (Pune) and a rural (Sinhagad) location in India. J. Geophys. Res. D Atmos..

[38] Draxler, R. R. HYSPLIT_4 User’s Guide. *NOAA Air Resources Laboratory* 1–38 (1999).

[39] Petit JE, Favez O, Albinet A, Canonaco F (2017). A user-friendly tool for comprehensive evaluation of the geographical origins of atmospheric pollution: Wind and trajectory analyses. Environ. Model. Softw..

[40] Taubman BF (2006). Aircraft vertical profiles of trace gas and aerosol pollution over the mid-Atlantic United States: Statistics and meteorological cluster analysis. J. Geophys. Res. Atmos..

[41] Brimblecombe P, Clegg SL (1988). The solubility and behaviour of acid gases in the marine aerosol. J. Atmos. Chem..

[42] Brimblecombe, P. *Air Composition and Chemistry*. (Cambridge University Press, 1986).

[43] Miller RL, Bradford WL, Peters NE (1988). Specific Conductance: Theoretical Considerations and Application to Analytical Quality Control. United States Geol. Surv. Water-Supply Pap..

[44] Beiderwieden E, Wrzesinsky T, Klemm O (2005). Chemical characterization of fog and rain water collected at the eastern Andes cordillera. Hydrol. Earth Syst. Sci. Discuss..

[45] Wbs, I. D. ASCAT Verification, Calibration & Validation Plan. 1–27 (2011).

[46] Verspeek J (2010). Validation and Calibration of ASCAT Using CMOD5.n. IEEE Trans. Geosci. Remote Sens..

[47] Anderson C, Figa-Saldaña J, Wilson JJW, Ticconi F (2017). Validation and Cross-Validation Methods for ASCAT. IEEE J. Sel. Top. Appl. Earth Obs. Remote Sens..

[48] Randles CA (2017). The MERRA-2 Aerosol Reanalysis, 1980 Onward. Part I: System Description and Data Assimilation Evaluation. J. Clim..

[49] Buchard V (2017). The MERRA-2 aerosol reanalysis, 1980 onward. Part II: Evaluation and case studies. J. Clim..

[50] Wagner, A. M. *et al*. Validation report of the CAMS near-real time global atmospheric composition service System evolution and performance statistics, 10.24380/dg9c-pm41 (2019).

[51] Inness A (2019). The CAMS reanalysis of atmospheric composition. Atmos. Chem. Phys..

[52] Duruisseau F, Huret N, Andral A, Camy-Peyret C (2017). Assessment of the ERA-Interim Winds Using High-Altitude Stratospheric Balloons. J. Atmos. Sci..

[53] Swail VR, Cox AT (2000). On the use of NCEP-NCAR reanalysis surface marine wind fields for a long-term North Atlantic wave hindcast. J. Atmos. Ocean. Technol..

[54] Hess NCL (2016). The NCEP/NCAR 40-Year Reanalysis Project. Am. Meteorol. Soc..

[55] Gatz DF, Smith L (1995). The standard error of a weighted mean concentration-I. Bootstrapping vs other methods. Atmos. Environ..

