# Supplementary material for: Influence of dust and sea-salt sandwich effect on precipitation chemistry over the Western Ghats during summer monsoon
Source: Sci Rep. 2019 Dec 16;9:19171. doi: 10.1038/s41598-019-55245-0 (PMC6915755; doi:10.1038/s41598-019-55245-0)
Supplement: Supplementary file 1 — Supplementary information [file 41598_2019_55245_MOESM1_ESM.pdf]

Influence of dust and sea-salt sandwich effect on precipitation chemistry over Western Ghats during summer monsoon

L.Yang[1], S.Mukherjee[1], G. Pandithurai[1*], V.Waghmare[1], P.D Safai[1]
[1]Indian Institute of Tropical Meteorology, Pune, India
Correspondence: [1*]pandit@tropmet.res.in

Supplementary Figures

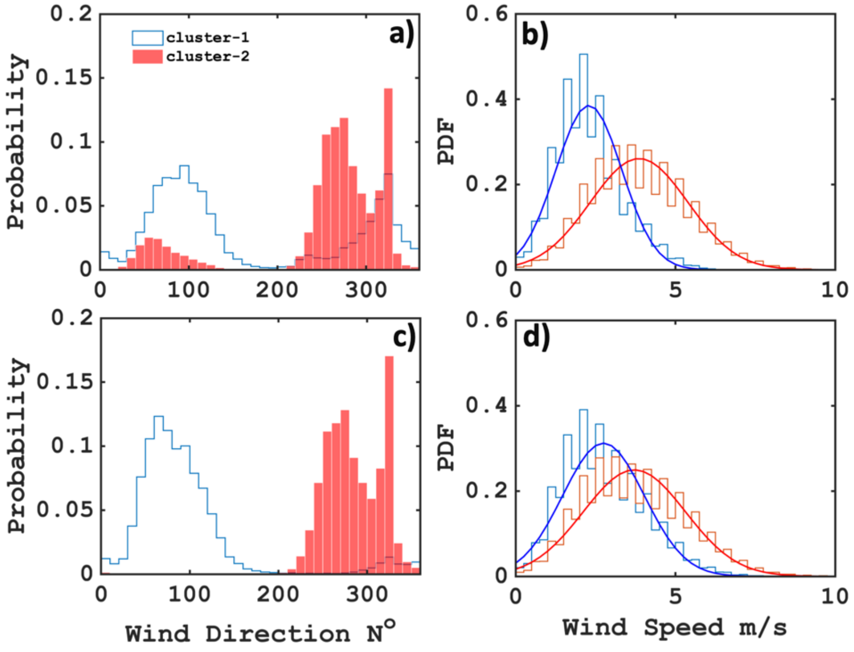

Supplementary Figure 1. Hourly HYSPLIT backward trajectories segregated by *k*-mean clusters, normalized probability and PDF (probability density function) histogram plot for local AWS wind direction **(a)** and wind speed **(b)**. And *k*-mean clustered with only local winds and no HYSPLIT segregation **(c, d)**.

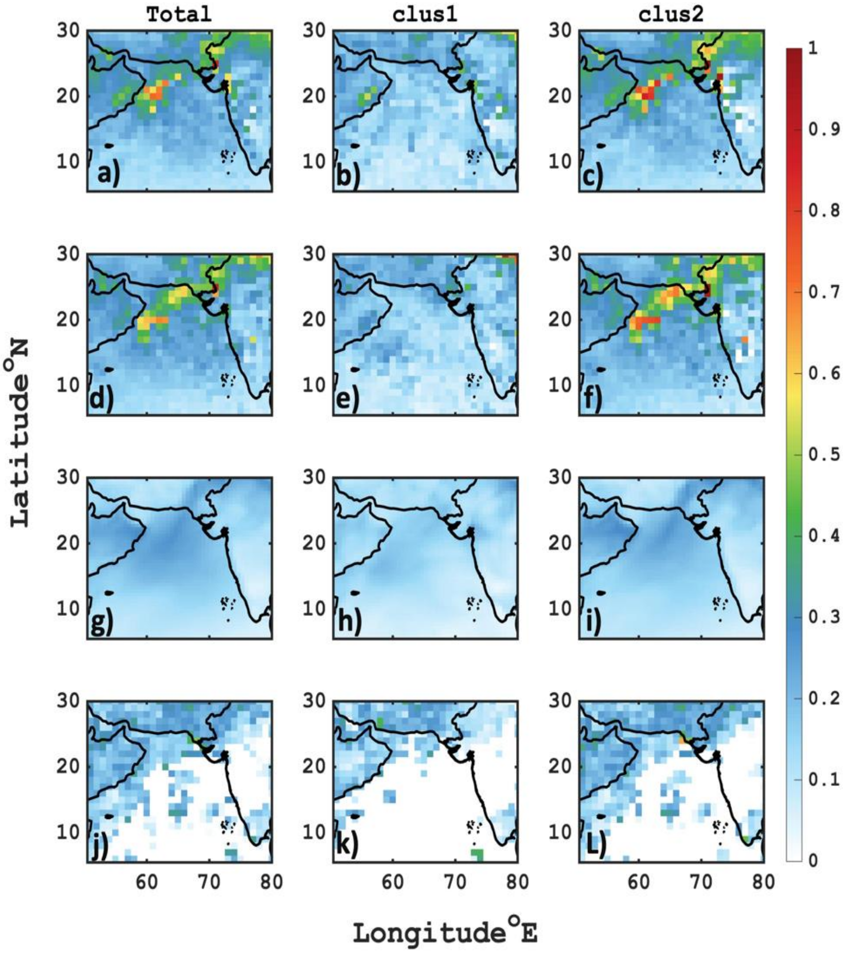

Supplementary Figure 2. Spatial variation of standard deviation of mean AOD at ~550nm for each grid point from MODIS-Terra **(a-c)**, Aqua **(d-f)**, MERRA2 **(g-i),** and OMI-Aura **(j-L)** over Arabian Sea during 2016 summer monsoon period.

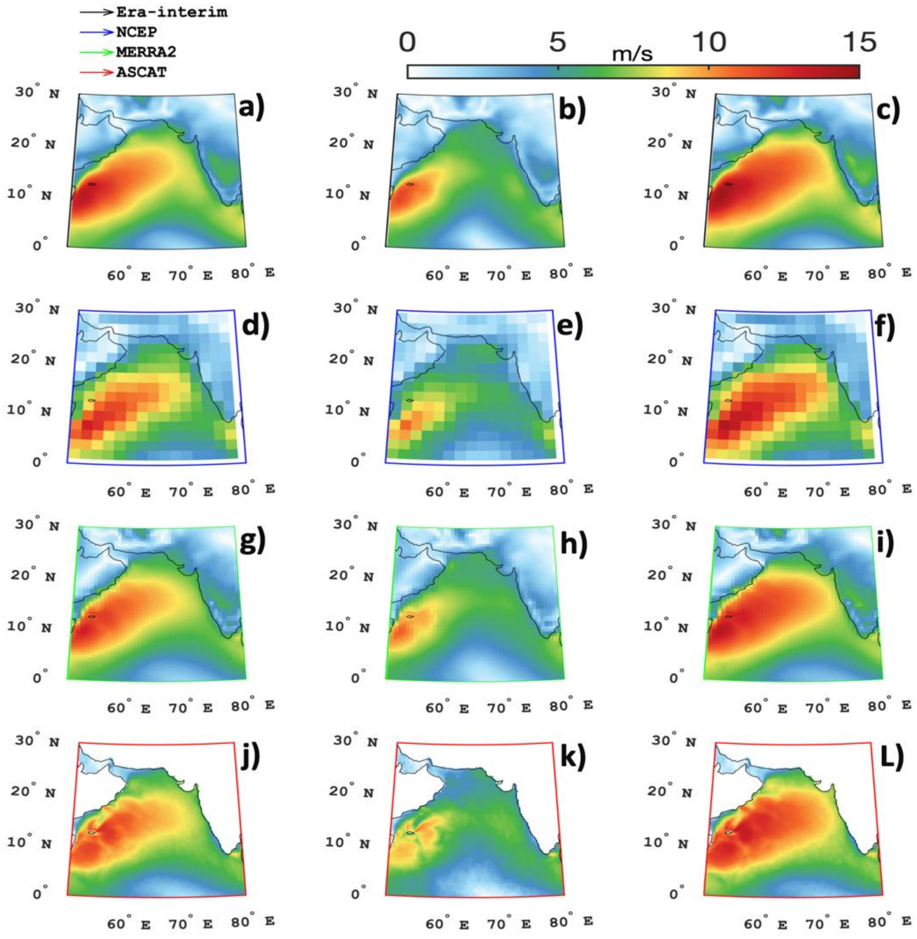

Supplementary Figure 3. Intercomparison between spatial pattern of Total (first column), cluster-1 (second column), and cluster-2 (third column) wind speed from different reanalysis and satellite based observation. The black outlined row represents Era-Interim **(a-c)**, blue outline depicts NCEP **(d-f)**, green stands for MERRA2 **(g-i)** and red denotes METOP-ASCAT satellite observation.

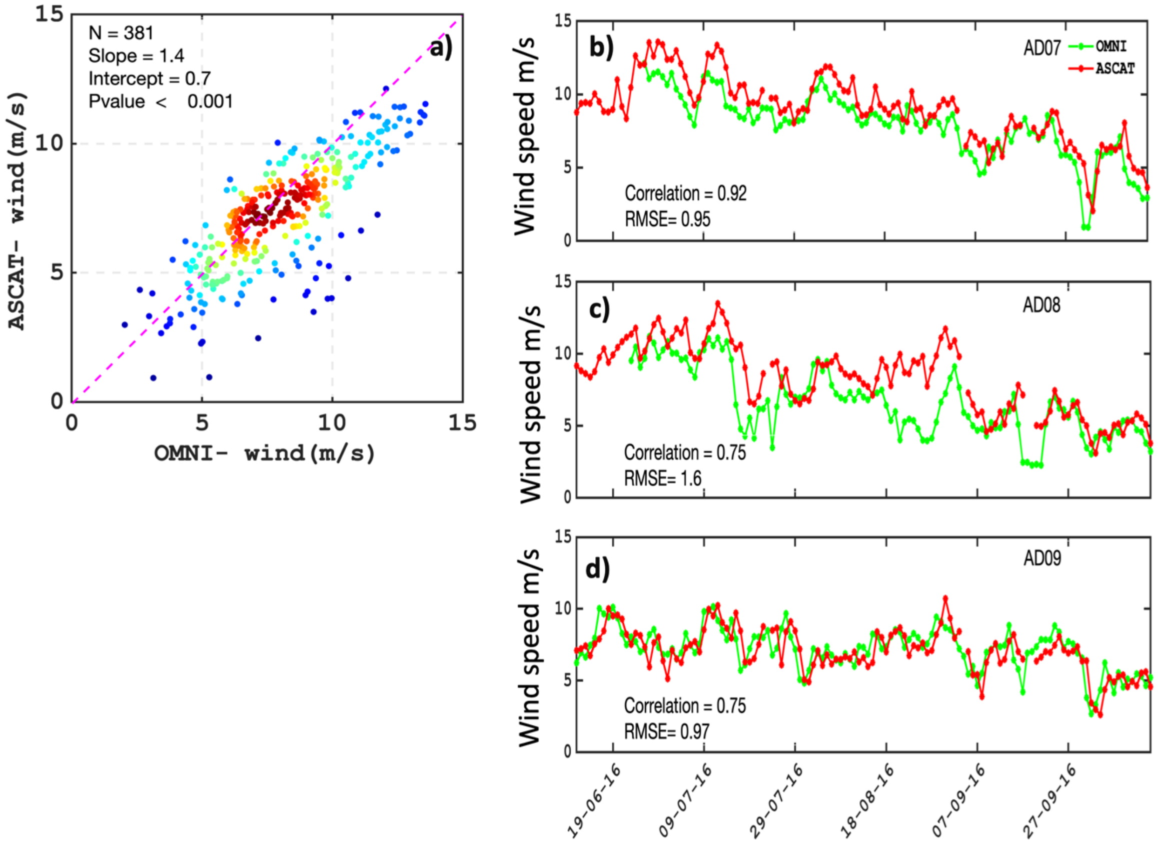

Supplementary Figure 4. Kernel density scatter plot **(a)** of satellite obtained ASCAT wind speed (m/s) vs cumulative OMNI buoy (AD07, AD08, and AD09) wind speed (m/s). Intercomparison between daily wind speed from individual OMNI buoy-AD07 **(b)**, AD08 **(c)** and AD09 **(d)** with nearest ASCAT grid point.

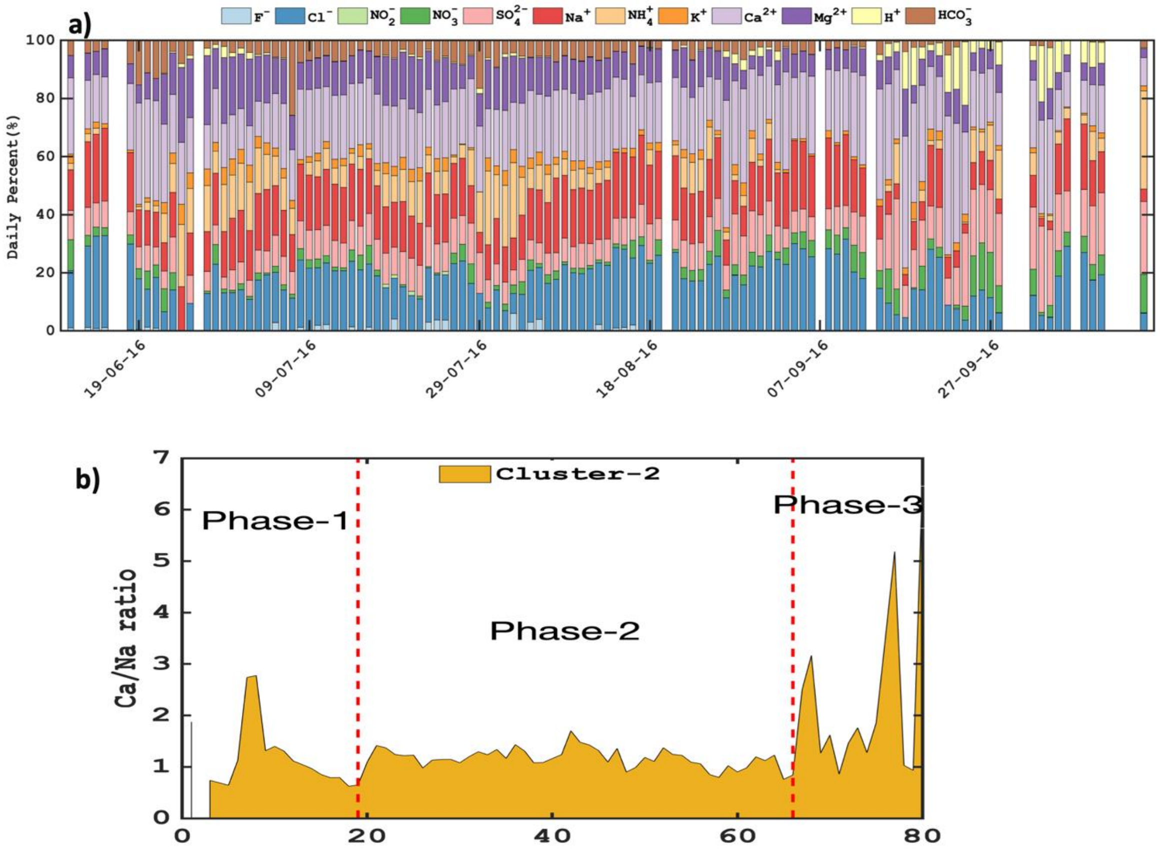

Supplementary Figure 5. Daily percentage of inorganic aerosols ionic concentration in rainwater samples **(a)**, and Ca^{2+} to Na^{+} ratios in cluster-2 during active phase-1, phase-2 and phase-3 time period. The red dashed demarcations divides phase-1, 2 and 3.

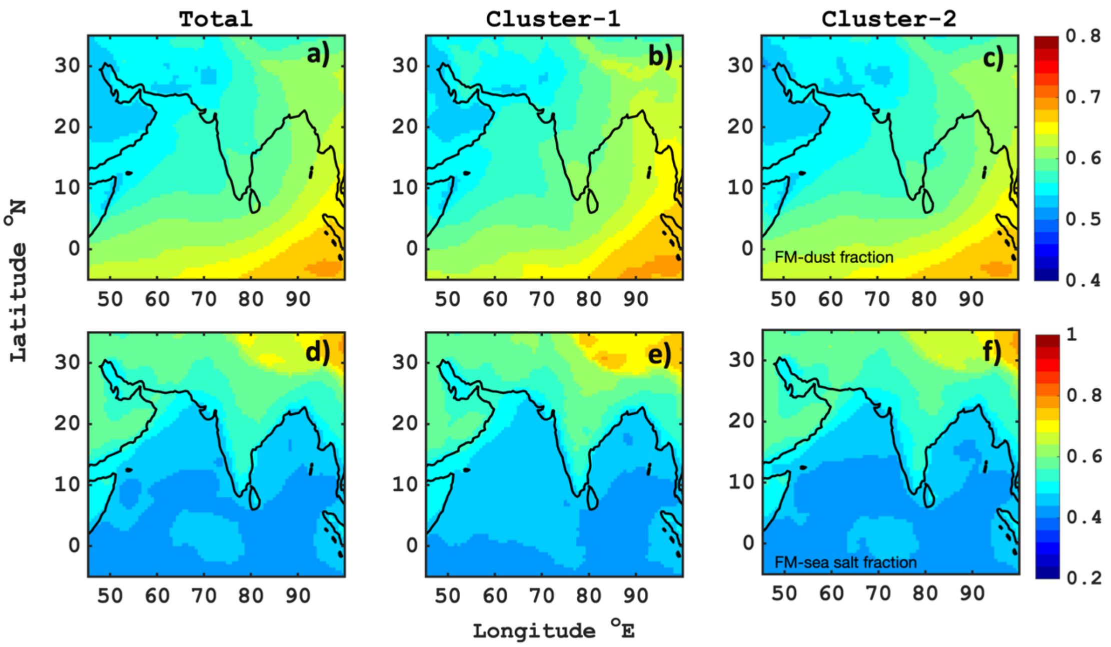

Supplementary Figure 6. Clustered fine mode fraction (PM2.5) of dust **(a-c)** and sea salt aerosols **(d-f)** over Indian Ocean during 2016 summer monsoon obtained from MERRA2 reanalysis.

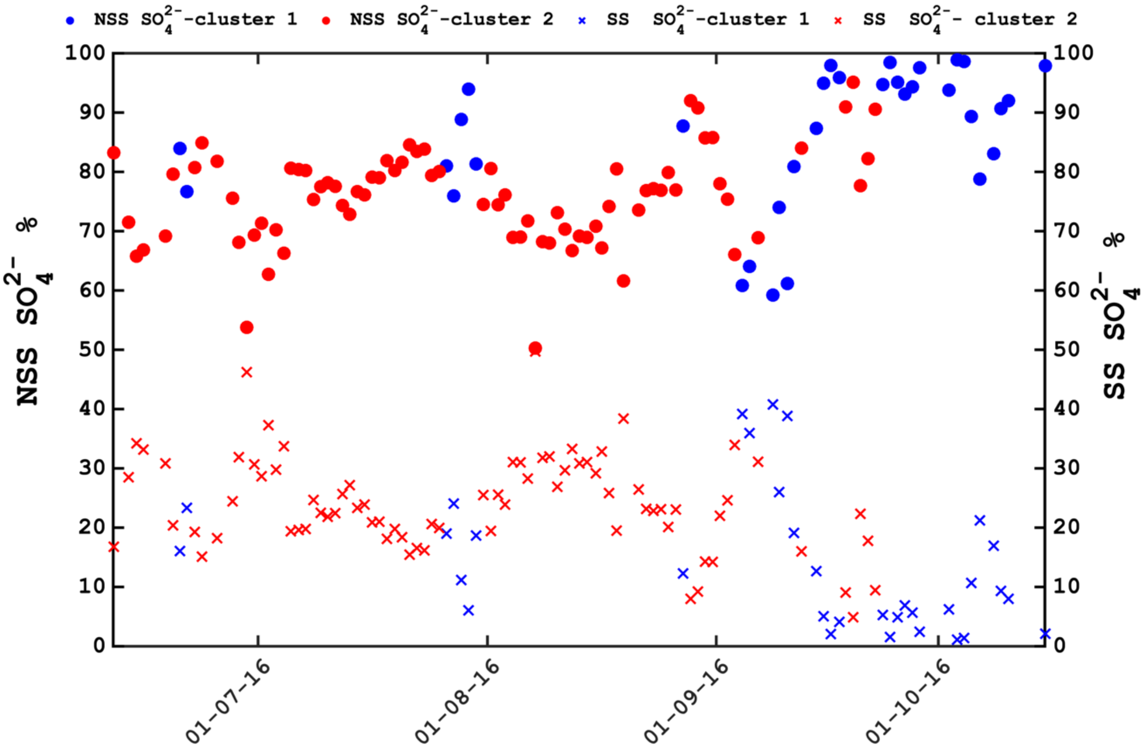

Supplementary Figure 7. Time series of percentage of NSS (Non-Sea Salt) SO_4^{2-} and SS (Sea Salt) SO_4^{2-} aerosols in daily collected summer monsoon (2016) rainwater samples for cluster-1 (blue) and cluster-2 (red).

$$SS\ SO_4^{2-}\% = \frac{(0.121\ \mathrm{X}\ Na^{+}_{rainwater})}{SO^{2-}_{4\ rainwater}} \quad (1)$$

$$NSS\ SO_4^{2-}\ \% = \frac{SO^{2-}_{4\ rainwater} - (0.121\ \mathrm{X}\ Na^{+}_{rainwater})}{SO^{2-}_{4\ rainwater}} \quad (2)$$

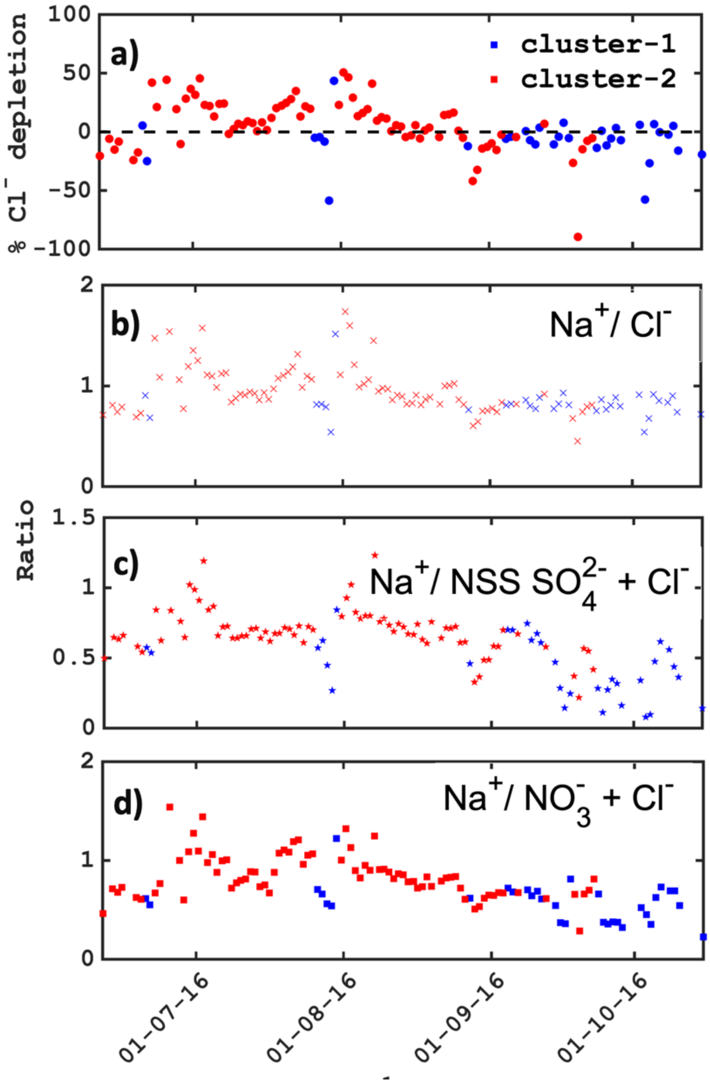

Supplementary Figure 8. Cl^- depletion percentage **(a)** in rainwater sample for monsoon 2016 and ratio of Na^+/ Cl^- **(b),** Na^+/(NSS SO_4^{2-}+Cl^-) **(c)** and Na^+/(NO_3^-+Cl^-) **(d)**.

$$Cl^-_{depletion\%} = \frac{(1.17[Na^+]_{rainwater\ aerosols} - [Cl^-]_{rainwater\ aerosols}}{1.17[Na^+]_{rainwater\ aerosols}} \qquad (3)$$

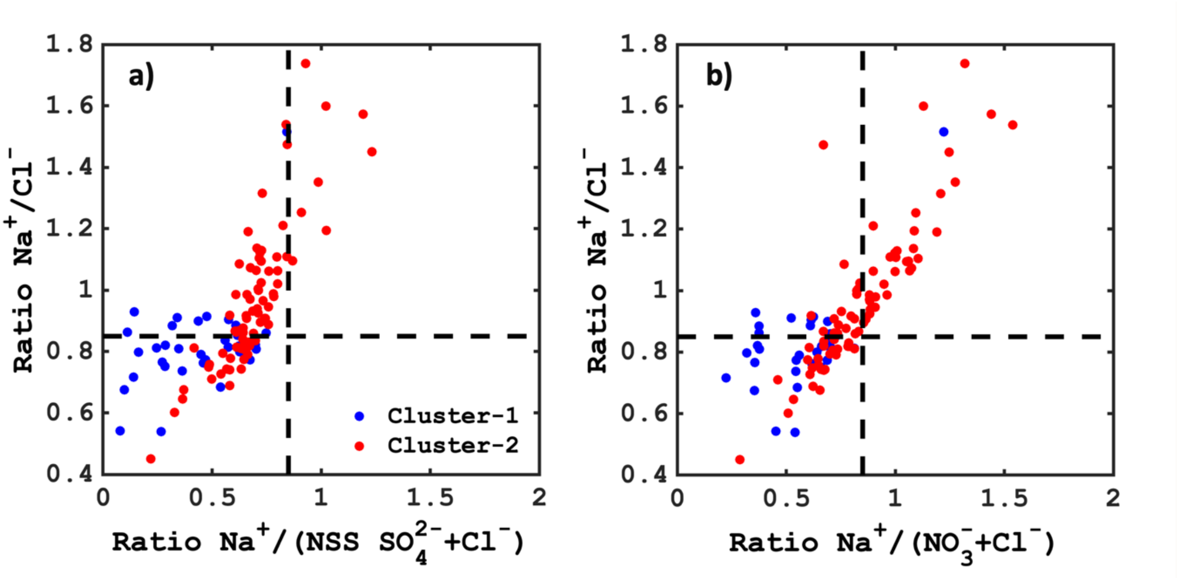

Supplementary Figure 9. Phase space plot of cluster-1 and cluster-2 ratios of Na^+/Cl^- vs $Na^+/(NSS\ SO_4^{2-}+Cl^-)$ **(a)** and Na^+/Cl^- vs $Na^+/(NO_3^-+Cl^-)$ **(b).** The dashed line indicates 0.85 ratio for x and y axis, that is representative for treated and untreated Cl^- depletion in rainwater samples.

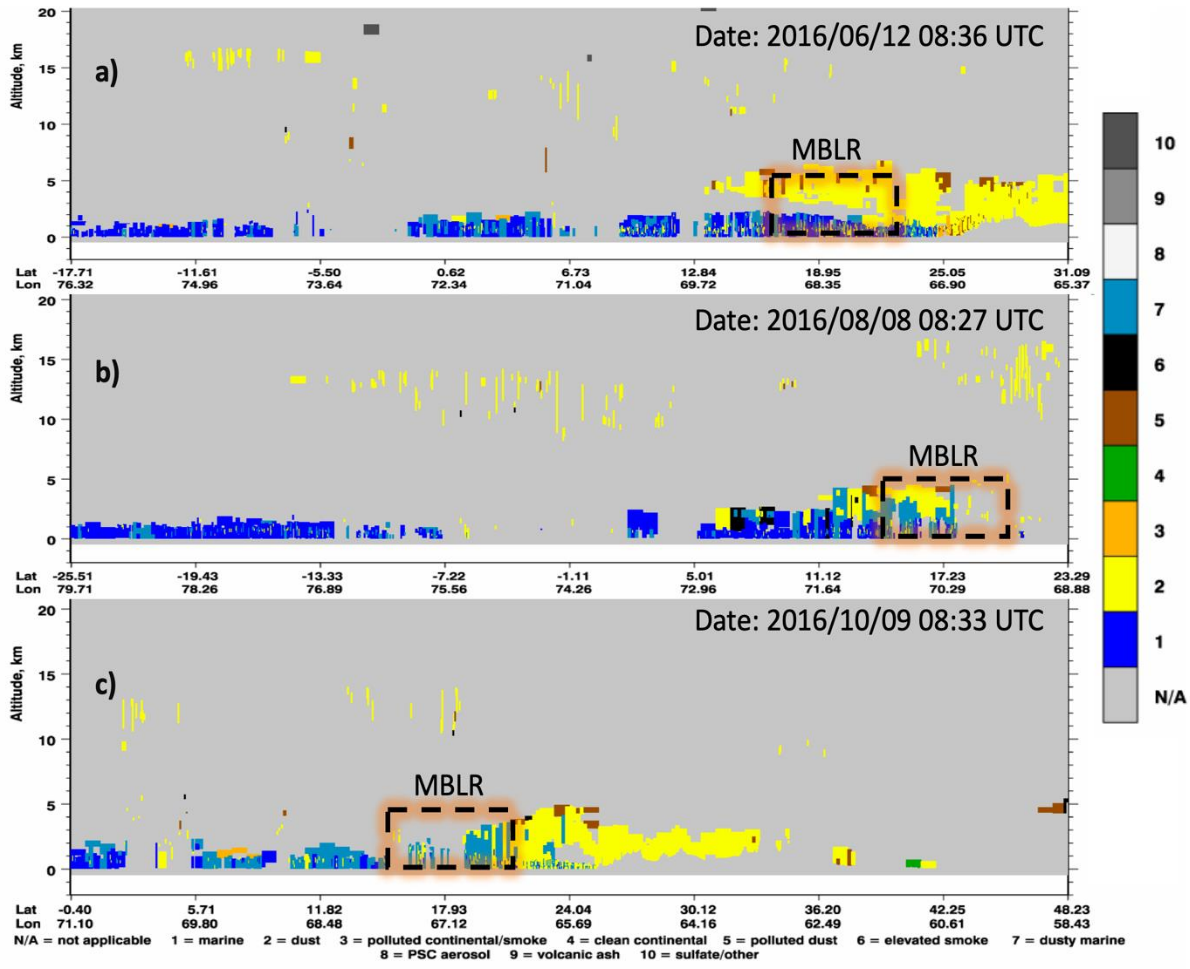

Supplementary Figure 10. Calipso LiDAR classified aerosol layer at initial **(a)**, active **(b)** and departure phase **(c)** of summer monsoon, 2016. The black dashed box with orange glow represent closest latitudinal vicinity of Mahabaleshwar (MBLR) near to the Western Ghats coast.

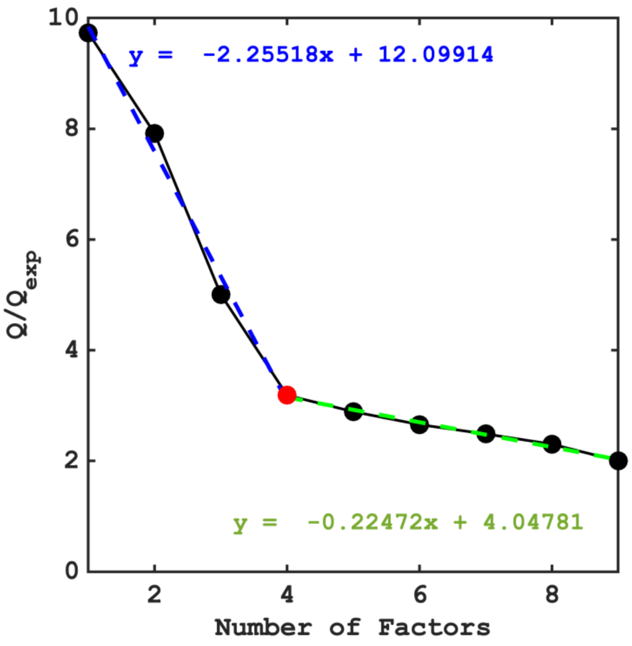

Supplementary Figure 11. Ratio of Q/Q_{exp} vs increasing number of factors from EPA-PMF analysis for summer monsoon rainwater sample 2016. Red dot circle represents optimum factor (4) chosen for the study period.

The choices of number of factors are based on error estimation method of reiterative runs with different number of factors[1] starting from factor 1 up to factor 9. The Q and Q_{exp} = (total number of non-weak data values in X sample matrix) − (numbers of elements in source matrix G and factor matrix F) was computed for each run by increasing a number of factor in each iteration. The ratio of Q/Q_{exp} was computed for increasing number of factors and minimizing the ratio of Q objective function that is the sum of the squared scaled residuals (i.e., Q_{true}) divided by overall Q_{exp} divided by number of non-weak species. The increasing factors, where the ratio it ought to not minimize Q function any further with constant slope value converging to zero slope was taken as an optimum factor solution for our PMF analysis as shown in the Figure 6 below. The slope value of Q/Q_{exp} ratio for number of factors from 1 to 4 was noticed with drastic reduction with -2.2 (Suppl. Figure 11)with intercept at 12, whereas after increasing beyond 4 factors the change in Q/Q_{exp} ratios was observed with about drop in ten folds of slope reaching to 0 (-0.2) depicting stabilization of factors beyond 4 factors.

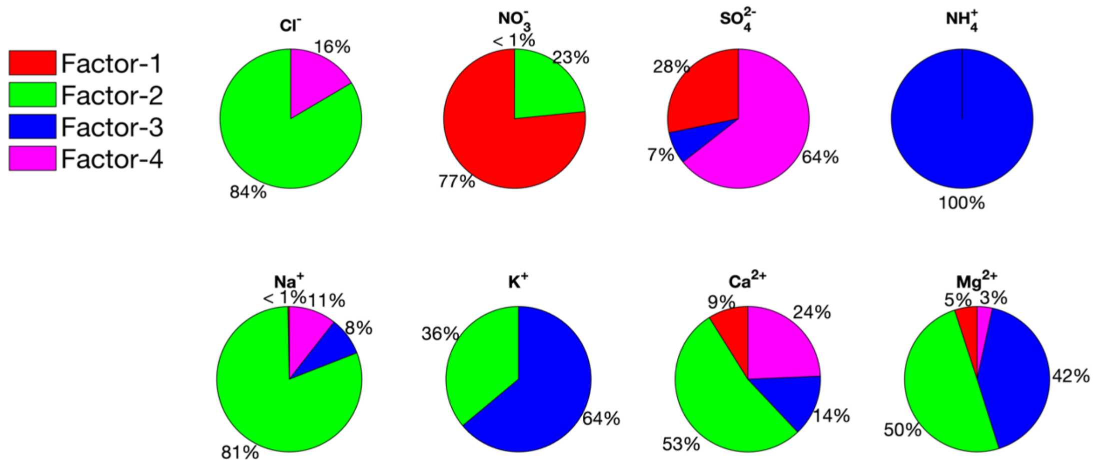

Supplementary Figure 12. PMF percentage of factor (Factor-1, Factor-2, Factor-3 and Factor-4) contribution to each rainwater species(Cl^-, NO_3^-, SO_4^{2-}, NH_4^+, Na^+, K^+, Ca^{2+}, and Mg^{2+}) during 2016 monsoon rainfall.

Suppl. Figure 12. shows the percentage contribution of each species to the respective factor. It can be clearly seen that the factor 1 was majorly dominated by the presence of SO_4^{2-} (28%) and NO_3^- (77%) which indicates the factor profile to be more associated with the secondary inorganic aerosol formation from the gaseous phase reaction of NO_x and SO_2 emitted from the anthropogenic sources. On the other hand, factor 2 is blend of both sea salt and dust aerosol as it is mostly contributed by the characteristic ions like Na^+ (81%), Cl^- (84%), Ca^{2+} (53%), and Mg^{2+} (50%).

Factor 3 is majorly dominated by the presence of K^+ (64%), NH_4^+ (100%) and Mg^{2+} (42%). Past studies have also shown that the biomass burning can contribute to both K^+ and NH_4^+ significantly[2,3]. And recent study for the same site Mukherjee et al., 2018[4] and Singla et al., 2019[5] have documented the importance and impact of wood burning on the aerosol number size as well as mass variability during the study period. Thus, one of the major contributors of factor 3 could be biomass burning. Author would also like to agree on the reviewer's point that the factor 3 can be attributed to both biomass burning and fertilizer use as it contains the signal of Mg^{2+} in addition to K^+ and NH_4^+. On the other hand factor 4 is majorly composed of SO_4^{2-} and Ca^{2+} that is attributed as calcium neutralization factor. To justify that NO_3^- doesn't efficiently neutralise is due to less abundance of it in atmosphere as compare to SO_4^{2-}. If we look closely on to the fact that the abundance of SO_4^{2-} in rainwater is ~2-4 times (Figure 5.g in manuscript) higher than that of the NO_3^- which implies that availability of the SO_4^{2-} ions in the neutralisation process is much higher as compared to the NO_3^- ions. Moreover,

Mukherjee et al., 2018[4] have also shown in their study that the submicron aerosol phase SO_4^{2-} concentration in the ambient atmosphere is ~6 times higher than that of the NO_3^- concentration for the same monsoon (JJAS-2016) period.

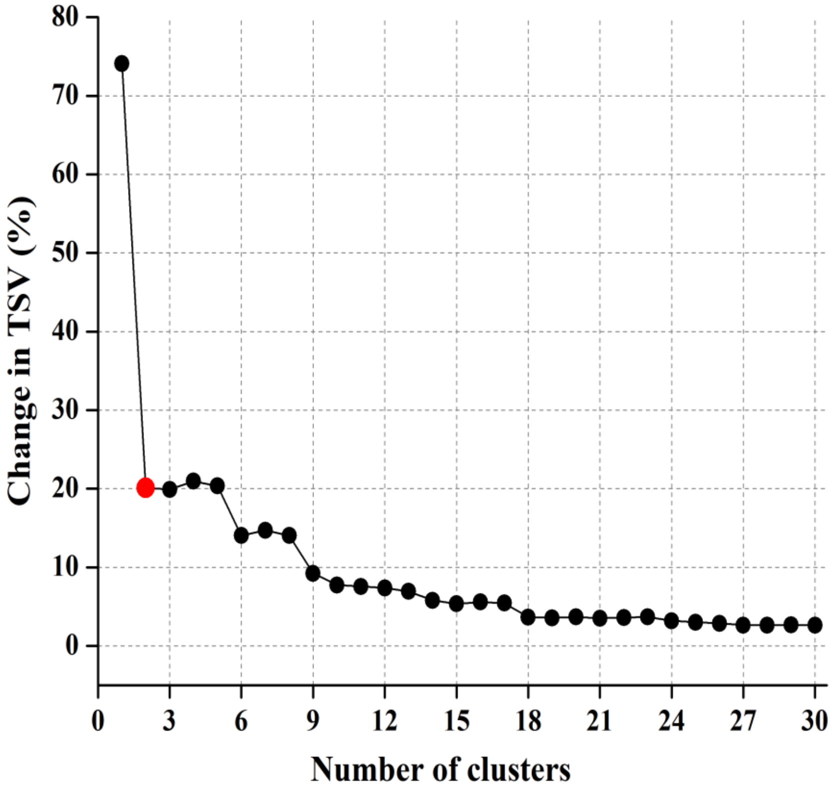

Supplementary Figure 13. HYPLIT backward trajectory, change in total spatial variance (TSV) for HACPL, Mahableshwar site during monsoon 2016. The red dot represents number (2) of cluster utilized in the study.

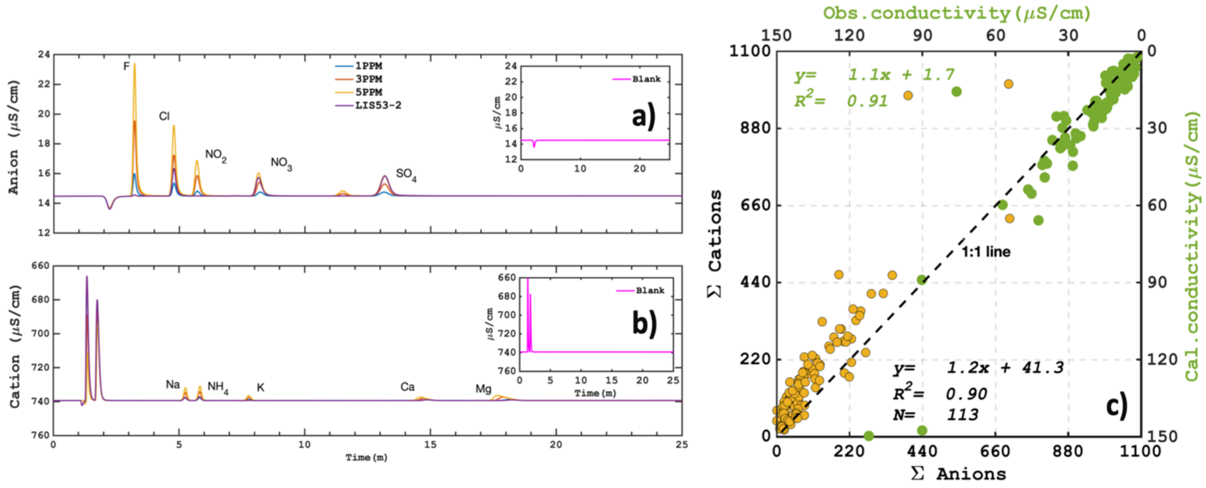

Supplementary Figure 14. IC (Ion chromatograph) calibration chromatogram for known concentrations at 1, 3, and 5 PPM for **(a)** Anions **(b)** Cations and Scatter plot of ionic balance (as shown by yellow dots) and comparison between calculated and observed conductivity **(c)** for 2016 rainwater samples (marked with green dots).

Supplementary References:-

1. Brown, S. G., Eberly, S., Paatero, P. & Norris, G. A. Methods for estimating uncertainty in PMF solutions: Examples with ambient air and water quality data and guidance on reporting PMF results. *Sci. Total Environ.* **518–519**, 626–635 (2015).
2. Bray, C. D. *et al.* Ammonia emissions from biomass burning in the continental United States. *Atmos. Environ.* **187**, 50–61 (2018).
3. Hegg, D. A., Radke, F. & Hobbs, P. V. Ammonia Emissions from Biomass Burning. *Geophys. Res. Lett.* **15**, 335–337 (1988).
4. Mukherjee, S. *et al.* Seasonal variability in chemical composition and source apportionment of sub-micron aerosol over a high altitude site in Western Ghats, India. *Atmos. Environ.* **180**, 79–92 (2018).
5. Singla, V., Mukherjee, S., Kashikar, A. S., Safai, P. D. & Pandithurai, G. Black carbon: source apportionment and its implications on CCN activity over a rural region in Western Ghats, India. *Environ. Sci. Pollut. Res.* **26**, 7071–7081 (2019).
